# A small-molecule carrier for the intracellular delivery of a membrane-impermeable protein with retained bioactivity

**DOI:** 10.1073/pnas.2407515121

**Published:** 2024-10-22

**Authors:** Xiqi Ma, Zhixiong Zhang, Andrea Barba-Bon, Dongxue Han, Zichun Qi, Baosheng Ge, Hua He, Fang Huang, Werner M. Nau, Xiaojuan Wang

**Affiliations:** ^a^College of Chemical Engineering, China University of Petroleum (East China), Qingdao 266580, China; ^b^School of Science, Constructor University, Bremen 28759, Germany

**Keywords:** lipid bilayer permeation, protein delivery, cytochrome C, chaotropic effect, boron clusters

## Abstract

Intracellular protein delivery has the potential to revolutionize cell-biological research and medicinal therapy, with broad applications in bioimaging, disease treatment, and genome editing. Herein, we demonstrate successful delivery of a functional protein, cytochrome c (CYC), by using a boron cluster anion as molecular carrier of the superchaotropic anion type (B_12_Br_11_OPr^2–^). CYC was delivered into lipid bilayer vesicles as well as living cells, with a cellular uptake ratio approaching 90%. Mechanistic studies showed that CYC was internalized into cells through a permeation pathway directly into the cytoplasm, bypassing endosomal entrapment. Upon carrier-assisted internalization, CYC retained its bioactivity, as reflected by an induced cell apoptosis rate of 25% at low dose (1 µM). This study furbishes a direct protein delivery method by a molecular carrier with high efficiency, confirming the potential of inorganic cluster ions as protein transport vehicles with an extensive range of future cell-biological or biomedical applications.

## Significance

The delivery of proteins into cells has been achieved by formulations based on liposomes, polymers, or nanoparticles. One limitation of these nanocarriers is that uptake proceeds through endocytosis with subsequent endocytic entrapment. This work introduces a simple molecular additive, a boron cluster anion, which allows direct permeation of cytochrome C as a target protein triggering apoptosis. The cluster forms a supramolecular complex with the protein, which permeates directly through the membrane into the cytosolic site of action. Because of the supramolecular interaction, the native protein is set free inside the cytosol, which ensures that cytochrome C remains active. This method circumvents the need for preparing encapsulating formulations because the boron cluster is highly water soluble and nonencapsulating.

Intracellular protein delivery is a transformative strategy in biological and pharmaceutical research (1, 2). The delivery of intrinsically impermeable proteins into living cells can replace poorly expressed, dysfunctional, or missing proteins and regulate key intracellular metabolic or signaling pathways (3). Accordingly, the method has broad application potential for bioimaging, cell and stem cell engineering, signaling pathway research, disease therapy, and genome editing (4–8). One clearly defined pharmaceutical potential of protein delivery is that even “undruggable” targets can be addressed (9) or that solid tumors can be targeted (10, 11). To affect intracellular protein delivery, any carrier approach needs to overcome numerous unfavorable intrinsic properties of most proteins, namely, poor membrane permeability, short half-life, large size, and susceptibility to degradation (12). Therefore, new effective intracellular delivery strategies are highly important in cell biology and medicine.

In response to demand, many techniques have been developed to deliver proteins into living cells (13). Physical-mechanical or so-called macro- and micromethods (14), such as microinjection (15–17), cell squeezing (18, 19), and electroporation (20–22), are the most efficient approaches for directly administering proteins to cells or extracellular vesicles, but their application range is restricted due to the low-throughput and disruptive characteristics (23) of these methods, which have very limited in vivo potential. Another commonly used method is to covalently fuse cargo proteins with cell-penetrating peptides (CPPs) (24, 25). However, in most cases, CPP-fused proteins enter the cell through endocytosis, during which they are captured in endosomes and unable to reach their target subcellular organelles to perform their desired biological functions (26). In addition, endosomally trapped proteins are routinely discharged by exocytosis or degraded by proteinases (27, 28), either resulting in very low cytoplasmic release of the delivered cargo (1%) (29) or requiring additional design concepts such as endosomal membrane disruption (30–32).

In recent years, much progress has also been made toward the use of microheterogeneous and nanocarrier-based delivery approaches (4, 6, 12, 23, 33–38). The corresponding formulations require the encapsulation of the cargo into liposomes (39) or virus-like particles (40) or their conjugation to inorganic (41) or polymeric (42, 43) nanoparticles. While these nanocarrier approaches allow tailoring of the formulations to a specific cargo type, endosomal entrapment of the assemblies remains a recurring interference (32, 44, 45) associated with the drawbacks of low delivery efficiency, decreased protein activity, or high toxicity. Finally, there are additional synthetically more elaborate approaches that are based on dynamic covalent bond formation to facilitate membrane passage, prominently those based on di- and oligosulfide-modified systems (46).

Molecular carrier approaches present an appealing alternative to the use of nanoscale formulations because they can pave the way for directing membrane permeation pathways into the cytosol; this approach is highly desirable for intracellular activity, as it allows continuous, endocytosis-independent migration into subcellular organelles or the nucleus (47). However, very few examples of molecular carriers for peptidic cargos are known, and the reported ones have traditionally involved amphiphilic molecules such as pyrene butyrate (48–51) or macrocyclic cyclodextrin (52, 53) or calixarene (54–58) derivatives, which have limited cargo scope because they are applicable only to cationic peptides of the cell-penetrating type rather than native proteins (55, 59). Recently, inorganic boron cluster (IBC) anions of the dodecaborate (B_12_X_12_^2–^, X = H, Cl, Br, I) (60) or decaborate type (B_10_X_10_^2–^, X = H, Cl, Br, I) (61), and cobalt bis(dicarbollide) (62) as well as polyoxometalates (63) have been introduced as a new class of molecular carriers, which draw their carrier potential from their superchaotropic ionic nature rather than from an amphiphilic structural design. These boron clusters are chemically highly inert (64–66), poorly metal coordinating, and weakly basic inorganic anions (67–69) which have already found numerous applications in different areas (64, 70–75), including therapy (65, 76–78). Due to the recently recognized superchaotropic nature of large cluster ions (69, 79–88), they exhibit unexpectedly high affinities for both proteins and membranes, which can be traced back to the chaotropic effect (60, 89). The chaotropic effect presents a generic driving force that accounts for the intrinsic affinity of chaotropic ions for organic interfaces and surfaces, including those of polymers, peptides, proteins, and concave macrocyclic binding sites (82, 83, 89, 90). Most important with respect to delivery applications is their own ability to interact with and permeate through lipid bilayer membranes (60, 91, 92). We have now applied the boron cluster approach to the delivery of a functional membrane-impermeable protein, cytochrome c (CYC, MW ca. 12 kDa). CYC is a positively charged protein that has evolved as a key protein target in delivery studies (93–95), owing to its potential in tumor therapy by inducing apoptosis (96).

## Results

### Intermolecular Interactions between IBC-Pr and CYC.

**Fig. 1.**
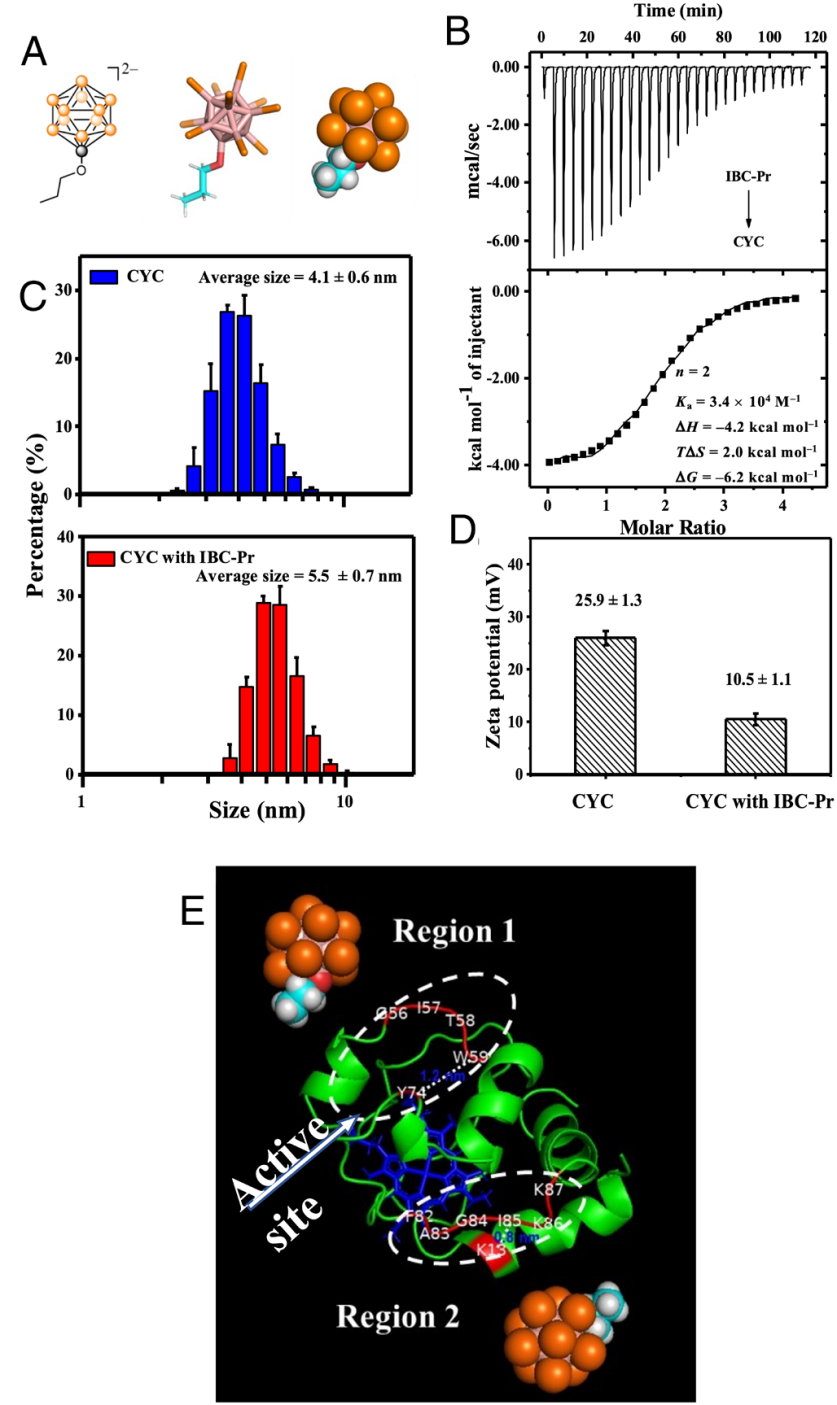
Intermolecular interactions between IBC-Pr and CYC. (*A*) Chemical-structural representations (orange represents boron-bromine bonds or bromine atoms) of IBC-Pr. (*B*) Microcalorimetric titration of CYC with IBC-Pr. Raw ITC data (*Top*) for sequential injections of IBC-Pr into the CYC solution and apparent reaction heats obtained from the integration of the calorimetric traces (*Bottom*). IBC-Pr/CYC concentrations in mM: 5/0.25. The stoichiometric *n* value for a one-set-of-sites model was found to be 2.0 ± 0.1. 10% error applies for *K*_a_ and ±0.5 kcal mol^–1^ for Δ*H*, *T*Δ*S*, and Δ*G* (quadruple replicates, on two instruments); fitting according to a sequential binding model with two different binding sites was equally possible, but because the thermochemical parameters in this fitting afforded similar values for both binding sites (Δ*H*_1_ = –3.9 ± 0.5 kcal mol^–1^ *versus* Δ*H*_2_ = –3.4 ± 0.5 kcal mol^–1^ and *T*Δ*S*_1_ = 2.5 ± 0.9 kcal mol^–1^ *versus T*Δ*S*_2_ = 2.2 ± 0.5 kcal mol^–1^), the fitted binding constants had larger errors (*K*_1_ = (8.5 ± 6.4) × 10^4^ M^–1^; *K*_2_ = (1.5 ± 0.3) × 10^4^ M^–1^). (*C*) Size distribution of CYC (1 µM) in the absence and presence of IBC-Pr (100 µM), measured by DLS; percentages derived from DLS intensities. (*D*) Zeta potential measurements of CYC (1 µM) in the absence and presence of IBC-Pr (100 µM). (*E*) Analysis of two binding regions (dashed circles); note that the heme ferric active site (shown in blue) remains accessible.

Among the transport-active inorganic clusters (60–63), we focused on the brominated dodecaborates, because they have the best balance of high activity and low cellular toxicity (61). Moreover, we selected B_12_Br_11_OCH_2_CH_2_CH_3_^2–^ (IBC-Pr, as sodium salt, Fig. 1*A*) rather than B_12_Br_12_^2–^, to demonstrate the potential of using monofunctionalized derivatives (97, 98). IBC-Pr was synthesized and purified as reported (97, 99, 100) and characterized via ^1^H NMR and ^11^B NMR spectroscopy as well as FTIR and ESI-HRMS measurements (*SI Appendix*, Fig. S1). The intermolecular interaction between IBC-Pr and CYC was quantified by isothermal titration calorimetry (ITC, Fig. 1*B*). The thermodynamic parameters showed that the binding is an enthalpically driven process, as expected for chaotropic interactions (83, 89), while the favorable entropic contribution may reflect an electrostatic component due to desolvation of ionic residues on the protein (101, 102). The interaction stoichiometry (*n* = 2.0 ± 0.1) suggested the formation of ternary 1:2 CYC/IBC-Pr complexes with two binding sites for boron clusters on the protein surface. Fitting by assuming a model with two identical binding sites afforded excellent agreement (Fig. 1*B*), such that the affinities to the two binding sites in CYC appear to be comparable. The resulting (average) binding constant to both sites was found to be sufficiently high, *K*_a_ = (3.4 ± 0.3) × 10^4^ M^–1^ or *K*_d_ = 29 µM, to ensure significant complex formation at high micromolar carrier concentrations but sufficiently low to ensure cargo release through a dynamic supramolecular equilibrium. In detail, at typical delivery concentrations of 1 to 10 µM CYC and 100 µM IBC-Pr, approximately 85% of CYC is expected to be complexed at equilibrium, and ca. 15% remains free.

The hydrodynamic size of the complex was measured by dynamic light scattering (DLS). The theoretical three-dimensional parameters calculated from their (native) chemical structure are ca. 1.0 × 0.7 × 0.7 nm^3^ for IBC-Pr and 2.6 × 3.2 × 3.3 nm^3^ for CYC (103). DLS revealed an average hydrodynamic diameter of 4.1 ± 0.6 nm for CYC alone and a distinctly larger one of 5.5 ± 0.7 nm for CYC with IBC-Pr (Fig. 1*C*), consistent with complex formation. Zeta potential measurements (Fig. 1*D*) showed that the binding of IBC-Pr reduced the positive surface potential of CYC from 25.9 ± 1.3 mV to 10.5 ± 1.1 mV. Chemical structure analysis showed that CYC is a protein with 8 units of expected positive surface charge at physiological conditions (pH 7.4) (104). These are expected to be partially neutralized by the binding of two doubly negatively charged IBC-Pr cluster anions, consistent with the experimentally observed decrease in protein surface charge.

Superchaotropic anions can be characterized as being strongly hydrophilic and lipophilic at the same time, that is, they can be very water-soluble and still have a high affinity for binding to hydrophobic interfaces or cavities (69, 80, 89). The hydrophobicity and charge distribution on the surface of CYC were therefore analyzed via simulations to identify the likely binding sites. As shown in Fig. 1*E*, Region 1 contains a hydrophobic cavity near the amino acid residues G-56, I-57, T-58, W-59, K-60, and Y-74. The diameter of the cavity is approximately 1.2 nm (distance between Y-74 and W-59). Region 2 is near the amino acid residues K-13, F-82, A-83, G-84, I-85, K-86, and K-87 and is highly positively charged. The width of Region 2 is about 0.8 nm (distance between I-85 and K-13). Both concave sites are *i*) sufficiently large, *ii*) hydrophobic, and *iii*) positively charged, such that we assign them as preferred binding sites for IBC-Pr in our 1:2 binding model (*SI Appendix*, Fig. S2).

Experimentally, fluorescence spectra of CYC before and after IBC-Pr binding were also investigated. The intrinsic fluorescence of CYC is mainly derived from tryptophan, which can be excited at 280 nm and affords an emission with maximum at 350 nm (105). Interestingly, the binding of IBC-Pr to CYC resulted in a distinct enhancement of the microenvironmentally responsive Trp fluorescence intensity and, counterintuitively, a slightly decreased average lifetime (from 2.05 to 1.99 ns, *SI Appendix*, Fig. S3). This opposing trend translates to an increased radiative decay rate in the presence of IBC-Pr (106, 107), which in turn is consistent with the relocation of at least one Trp residue, likely W-57 in Region 1, into a more polarizable environment, that is, near the highly polarizable perbrominated boron cluster (61, 69, 89). The fluorescence response was consistent with the molecular modeling analysis.

### Structural and Functional Integrity of CYC in the Presence of IBC-Pr.

**Fig. 2.**
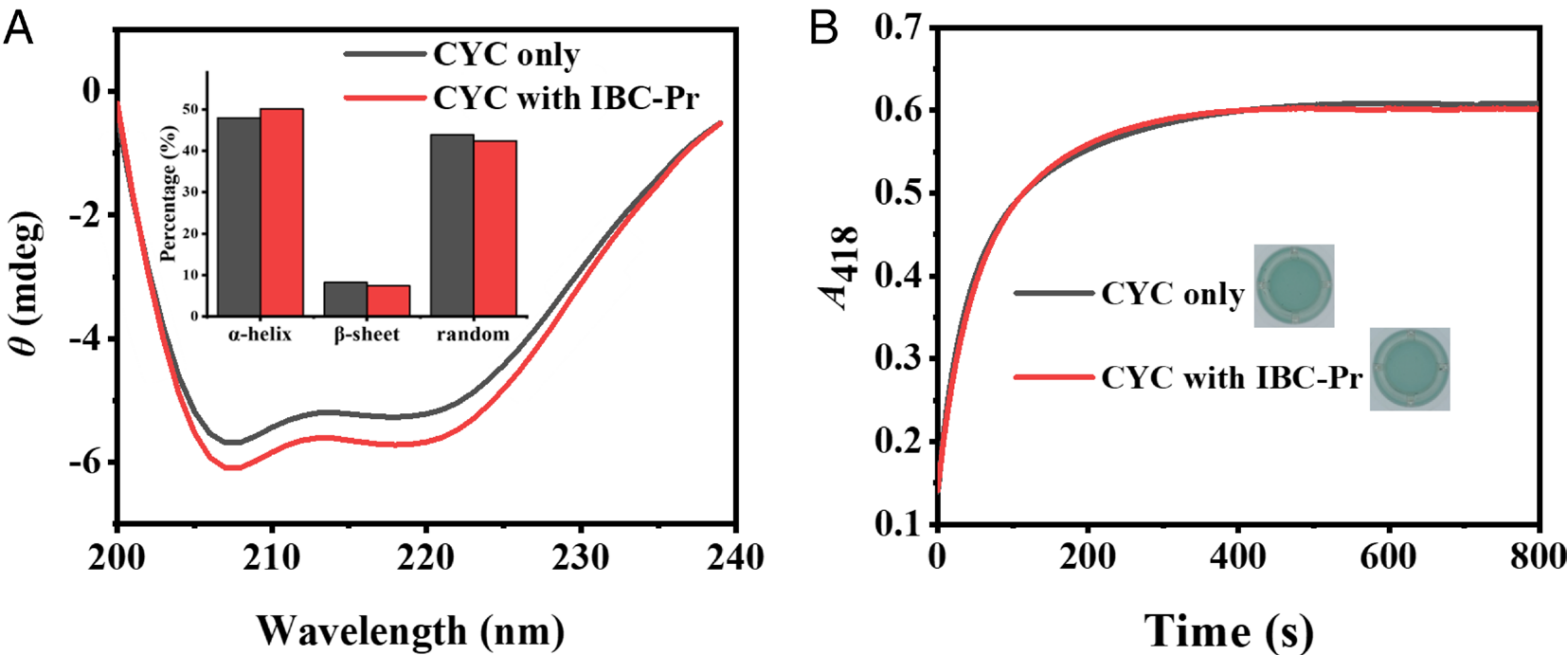
Structural and functional integrity of CYC. (*A*) CD spectra of CYC (1 µM) in the absence and presence of IBC-Pr (100 µM). In the inset, the calculated percentage of secondary structure contributions is shown (*Methods*), suggesting minimal conformational change upon binding of IBC-Pr. (*B*) Kinetics of peroxidase activity of CYC (10 µM) in the absence and presence of IBC-Pr (100 µM); the visual formation of the green radical cation (ABTS^⋅+^) after 20 min is shown in the inset. The presence of serum (10% (v/v) FBS) had no significant effect on the enzyme kinetics.

The influence of IBC-Pr binding on the structure and activity of CYC was investigated by circular dichroism (CD) spectroscopy and enzymatic assays. As shown in Fig. 2*A*, there was only a subtle change in the secondary structure of CYC in the presence of IBC-Pr (an achiral additive), with a deviation of 2%. Similarly, neither a shift in *λ*_max_ of the CYC fluorescence emission (*SI Appendix*, Fig. S3) nor changes in the UV absorption spectra (108) of CYC were observed in the presence of IBC-Pr (*SI Appendix*, Fig. S3). These results confirmed the retention of the native conformation of the protein at large.

Regarding the retention of bioactivity, CYC not only is an apoptosis-initiating protein (see below) but concomitantly possesses peroxidase activity, which is typically exerted in the mitochondrial respiratory chain. This is exploited in enzyme assays, in which the ability of CYC to catalyze the oxidation of 2,2’-azino-bis(3-ethylbenzthiazoline-6-sulfonate) (ABTS) to its green radical cation (ABTS**^⋅+^**) (109) can be monitored by UV spectrophotometry at 418 nm. As depicted in Fig. 2*B*, the kinetic traces of the CYC-catalyzed reactions were virtually superimposable in the absence and presence of IBC-Pr, even if ca. 85% of CYC was complexed under those conditions. The combined data suggest that the interaction between IBC-Pr and CYC affects neither its structure nor its catalytic activity in a pronounced manner, that is, the CYC/IBC-Pr complex retains these functionally critical properties. It also does not cause any aggregation or precipitation, as both would invariably reduce activity, and neither were these observed by DLS experiments (see above).

### Delivery of CYC with IBC-Pr Through Artificial Membranes.

The advantage of molecular carriers is that their ability to transport cargo across the lipid bilayer can be directly quantified by established in vitro transport assays with model membranes. This allows one to obtain evidence for carrier-assisted lipid-bilayer permeation of the cargo as well as to identify the most suitable working concentrations, information which needs to be empirically assessed for microheterogeneous carrier formulations. As a rule, molecular carriers that exhibit transport of a particular cargo across model membranes are showing cellular activity as well, because the pertinent transport mechanism (direct permeation through the lipid bilayer) is omnipresent in cellular membranes, in addition to energy-dependent pathways (endocytosis).

**Fig. 3.**
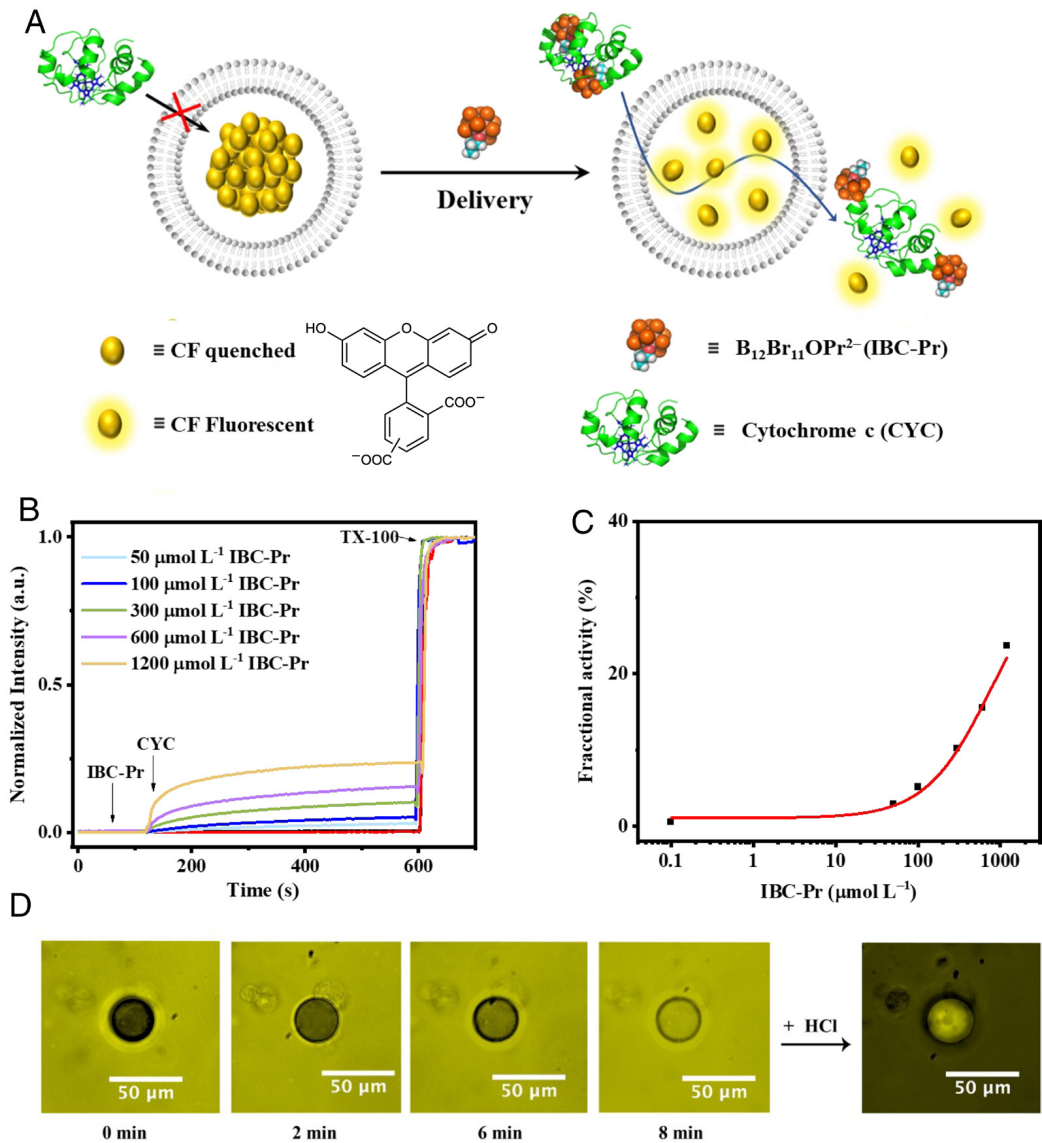
Delivery of CYC through artificial membranes. (*A*) Schematic representation of the CF assay. (*B*) Changes in CF emission (*λ*_ex_ = 492 nm, *λ*_em_ = 517 nm) in CF-encapsulated DMPE/DPPG/CHOL(1/2/1) liposomes as a function of time during the addition of different concentrations of IBC-Pr (0 to 1,200 μM) at *t* = 60 s, CYC (5 μM) at *t* = 120 s, and TX-100 at *t* = 600 s, for calibration, and (*C*) fractional activity for IBC-Pr with corresponding Hill curve fit; note that the maximal concentration of IBC-Pr was limited by its membrane-lytic effect above ca. 1.2 mM. (*D*) Fluorescence microscopy images of EYPC giant vesicles after the addition of CYC-FITC (10 μM) and IBC-Pr (50 μM) recorded after different incubation times (0 to 8 min); note the efficient boron cluster-induced uptake of CYC-FITC into the giant vesicle and the subsequent quenching of extravesicular fluorescence by addition of acid (pH 5.0). See *SI Appendix*, Fig. S6, for controls with brightfield micrographs.

In experimental detail, the ability of IBC-Pr to deliver CYC directly through a phospholipid bilayer membrane was investigated in large unilamellar vesicles (ca. 120 diameter, *SI Appendix*, Fig. S4) by using the carboxyfluorescein (CF) assay (Fig. 3*A*) (54). We selected 1,2-dimyristoyl-*sn*-glycero-3-phosphoethanolamine (DMPE)/1,2-dipalmitoyl-*sn*-glycero-3-phospho-(1’-rac-glycerol) (DPPG)/cholesterol (CHOL, 1/2/1 molar ratio) liposomes (60, 62) (Fig. 3*B*) as well as egg yolk phosphatidylcholine (EYPC) ones (*SI Appendix*, Fig. S5); the former have a better resemblance to cellular membranes due to their negative surface charge and presence of cholesterol. In a typical time-resolved fluorescence experiment, CF emission was monitored during the sequential addition of IBC-Pr (*t* = 60 s) and CYC (*t* = 120 s, Fig. 3*B*). The release of all the encapsulated CF by adding the surfactant Triton X-100 (TX-100, *t* = 600 s) allowed normalization of the fluorescence intensity data. The addition of only one of the components (IBC-Pr or CYC) did not produce any changes in the fluorescence of the system, suggesting that there was no membrane translocation of either cargo alone nor membrane disruption or other forms of dye efflux; moreover, the integrity of the vesicles in the presence of cargo and IBC-Pr (up to 1.2 mM) was confirmed by DLS experiments (*SI Appendix*, Fig. S4).

In the presence of IBC-Pr, delivery of CYC (5 μM) was signaled by a time-resolved increase in fluorescence of CF, which was expectedly dependent on the IBC-Pr concentration. To quantitatively characterize the transport efficiency, the normalized fluorescence response was plotted against the concentration of IBC-Pr, and the resulting dose–response curves were fitted according to the Hill equation to obtain the characteristic transport parameters, the maximal transmembrane activity (*Y*_max_), and the concentration needed to achieve 50% of *Y*_max_ (*EC*_50_) (56). Since the IBC-Pr concentration could not be studied above 1.2 mM due to incipient membrane disruption, the fitting results were approximate (*Y*_max_ ca. 35%; *EC*_50_ = 770 ± 80 μM, Fig. 3*C*) but sufficient to conclude that high micromolar concentrations of clusters induce fast (within 5 min) CYC uptake in model membranes. Empirically, for cellular experiments, the concentration of carrier can be adjusted at least 4 times below the vesicular *EC*_50_ because longer incubation times are used (on an hourly time scale) (60).

Strictly speaking, the CF assay does not provide direct evidence for CYC permeation, but signals it indirectly, by dye efflux. To directly visualize the lipid bilayer permeation of the protein, we also used larger, so-called giant vesicles of 50±25 μM diameter, which can be imaged by conventional fluorescence microscopy. For this purpose, CYC was labeled with fluorescein isothiocyanate (FITC), by-passing the need for other vesicular additives. Indeed, the experiments provided evidence that the fluorescently labeled protein was membrane-impermeable in the absence of carrier, but permeated effectively, within less than 10 min, in the presence of IBC-Pr, resulting in an almost equilibrated fluorescence intensity in bulk solution and inside the vesicles (Fig. 3*D*, *SI Appendix*, Fig. S6); subsequent addition of acid (HCl, pH 5.0) resulted in a selective quenching of the extravesicular phase, because the FITC chromophore is pH sensitive (110).

### Delivery of CYC with IBC-Pr through Cellular Membranes.

To test the transferability from vesicle models to biological membranes, intracellular CYC delivery with IBC-Pr as molecular carrier was studied in HeLa cells. First, to exclude any significant cytotoxicity of IBC-Pr in the concentration range required for transport, MTT assays were carried out. As shown in *SI Appendix*, Fig. S7, the physiological state of the cells was not affected when the IBC-Pr concentration was below 400 μM. Only at millimolar concentrations was a significant drop in HeLa cell viability observed (to 85%), consistent with the observation of membrane disruption in the vesicle experiments (above 1.2 mM). This is in line with the generally good biocompatibility of boron clusters (60, 70, 111), which have already found their way into clinical applications, e.g., for boron neutron capture therapy (BNCT) (65, 76–78). Accordingly, micromolar IBC-Pr concentrations should be sufficiently biocompatible to conduct intracellular protein delivery experiments (112).

**Fig. 4.**
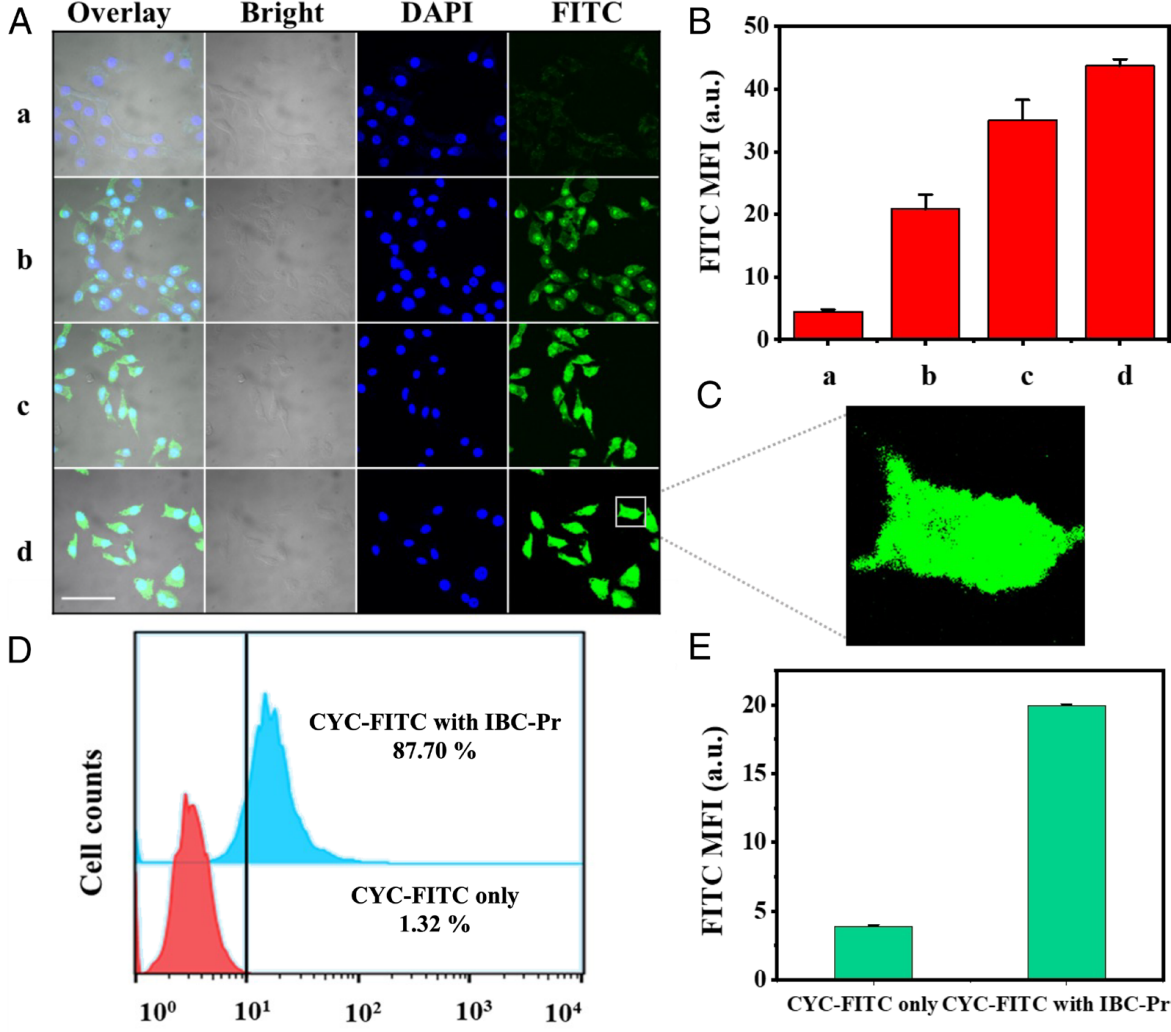
Delivery of CYC through cellular membranes. (*A*) Confocal images of HeLa cells after incubation with CYC-FITC (1 μM) and a) without or with b) 25 μM, c) 50 μM, or d) 100 μM IBC-Pr for 3 h; scale bar represents 50 μm. Representative images of three biological replicates. (*B*) Mean intensity of FITC fluorescence from the micrographs in panel (*A*). (*C*) Enlarged confocal image. (*D*) Flow cytometry and (*E*) quantitative analysis of the cytometry results for HeLa cells after incubation with 1 μM CYC-FITC and 100 μM IBC-Pr. Error bars indicate SD (*n* = 3).

For fluorescence staining, the labeled protein (CYC-FITC) was used. Living HeLa cells were incubated for 3 h with CYC-FITC alone or in an in situ mixture of CYC-FITC/IBC-Pr, subsequently fixed, and scanned by confocal laser-scanning microscopy. We maintained the protein concentration at 1 μM (well below its *IC*_50_ value for inducing long-term apoptosis, see below) and varied the concentrations of IBC-Pr from 25 to 100 μM, at the onset of transport activity in the model membranes (Fig. 3*C*), but well below its membrane-disrupting and cell-toxic concentrations. As shown in Fig. 4*A*, CYC-FITC alone was unable to enter HeLa cells, and only very weak green fluorescence was detected in the cells after 3 h of incubation. In contrast, in the presence of IBC-Pr, the fluorescence signal of CYC-FITC became readily observable and increased with carrier concentration (Fig. 4*B*). These results are consistent with the in vitro findings with CYC, verifying the successful intracellular delivery of CYC-FITC by IBC-Pr. Enlarged images of HeLa cells incubated with a mixture of CYC-FITC and IBC-Pr further revealed that the internalized CYC-FITC was located evenly within the cells, also in the cytoplasm and nucleus (Fig. 4*C*). A similar cellular distribution was observed when we conducted experiments with live instead of fixed HeLa cells (*SI Appendix*, Fig. S8). We also transferred the method to another cell line, A549, and obtained comparable staining results (*SI Appendix*, Fig. S9). Additionally, the protein was labeled with carboxytetramethylrhodamine (TAMRA) as an alternative, less pH-sensitive chromophore (CYC-TAMRA), which afforded also the desirable uptake into the cytosol, albeit with a lower nuclear staining propensity (*SI Appendix*, Fig. S10). Important to note, while IBC-Pr was able to transport the positively charged CYC (pI = 9.6) (113), the negatively charged green fluorescent protein (GFP, pI = 6.2) (114) was not transported into cells (*SI Appendix*, Fig. S11). Similarly, the negatively charged bovine serum albumin (BSA, pI = 5.5) (115) had been previously found to be inactive in liposomal transport assays (60). This suggests that the negatively charged boron clusters, in the absence of other additives, display a selectivity for transporting positively charged proteins.

Flow cytometry was also used to quantitatively evaluate the intracellular delivery efficiency. As shown in Fig. 4*D*, after free CYC-FITC was incubated with HeLa cells, the cellular uptake ratio was almost negligible (1.3%). When the mixture of CYC-FITC with IBC-Pr was applied, the cellular uptake ratio increased to 88%. Quantitative analysis (Fig. 4*E* and *SI Appendix*, Table S1) showed that the average fluorescence intensity of live HeLa cells treated with the CYC-FITC/IBC-Pr mixture was at least five times greater than that of cells incubated with CYC-FITC alone, again signaling highly effective protein delivery. CYC transport by the boron cluster was found to be compatible with the presence of serum in the incubation media, which showed a similar uptake efficiency (*SI Appendix*, Table S1). As a control, we also tested Lipofectamine^TM^ 3000, a commercially available nanocarrier frequently used for nucleic acid transfection, but no cellular uptake of the protein was found (*SI Appendix*, Fig. S12). This demonstrates that boron clusters serve as complementary carriers with a distinct cargo scope.

Although labeling with FITC is a standard method for following the fate and action of CYC by fluorescence (93, 94, 101), the omnipresent question remains whether all or part of the observed FITC fluorescence stems from inactive proteins that may be denatured, misfolded, or unfolded when shuttling through the cellular membrane. In addition, hypothetically, FITC can be already cleaved off (hydrolytically or enzymatically) as free dye or peptide fragment from the protein, falsely implying the uptake and presence of intact CYC. Consequently, its biological activity needed to be validated (116).

### CYC Retains Its Intracellular Bioactivity After Delivery by IBC-Pr.

**Fig. 5.**
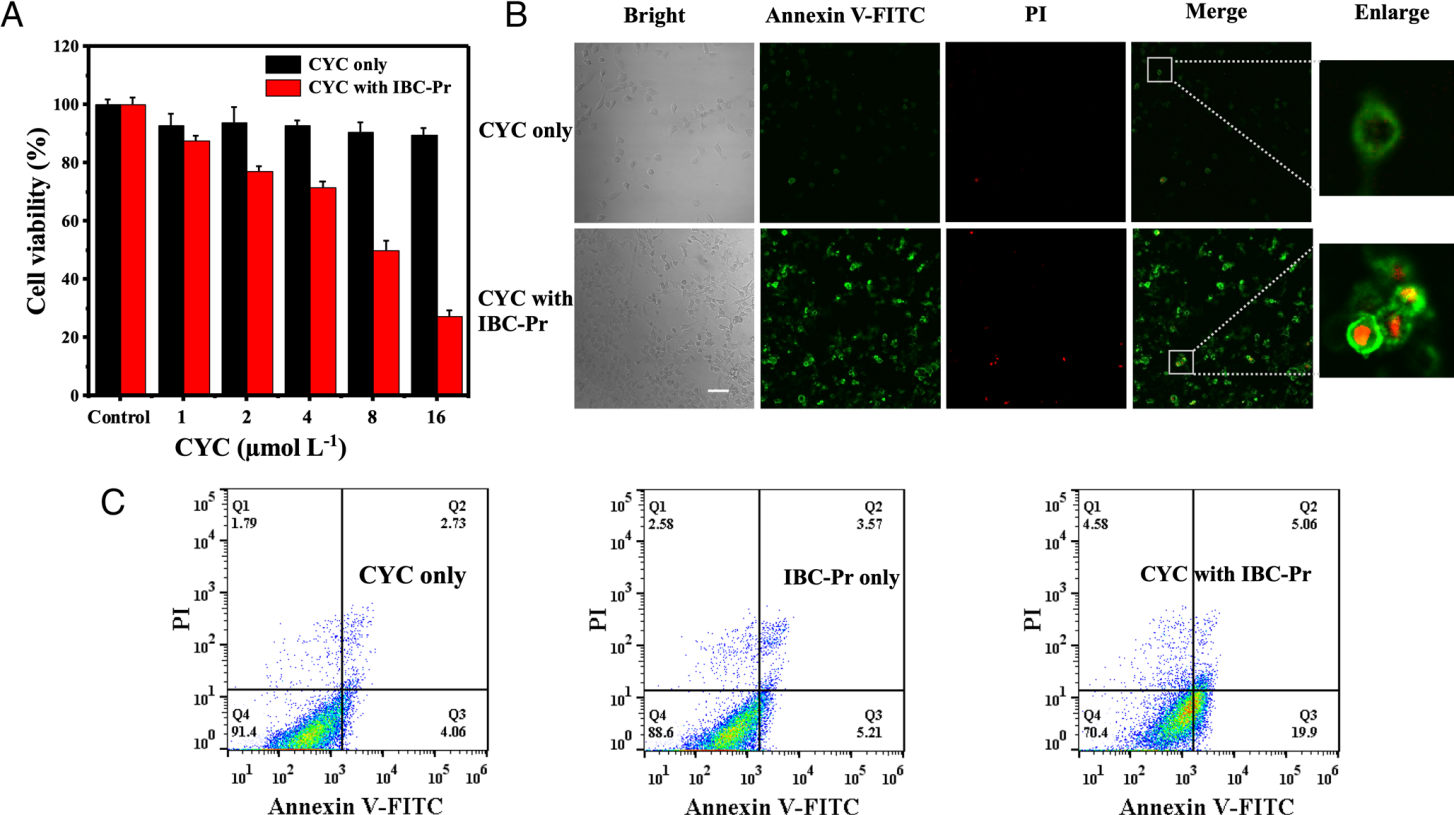
Cellular bioactivity of CYC after intracellular delivery by IBC-Pr. (*A*) Evaluation of the viability of HeLa cells treated with CYC (0 to 16 μM) with 100 μM or without IBC-Pr after 48 h of incubation. Error bars indicate SD (*n* = 3). (*B*) Confocal laser scanning microscopy images of HeLa cells after incubation with CYC (1 μM) in the absence or presence (100 μM) of IBC-Pr after 24 h of incubation followed by subsequent staining with Annexin V-FITC/PI; scale bar represents 50 μm. Representative images of three biological replicates are shown. (*C*) Flow cytometric apoptosis assay of HeLa cells after 24 h of treatment with CYC (1 μM), IBC-Pr (100 μM), or both. Q1: necrotic cells, Q2: late apoptotic cells, Q3: early apoptotic cells, and Q4: living cells. The numbers in the Q2 and Q3 areas indicate the percentage of late and early apoptotic cells, respectively.

CYC is a vital mediator of apoptosis that typically triggers the caspase activation cascade in the cytoplasm only after intracellular mitochondrial release (95, 117). Determining whether and to what degree any extracellularly internalized CYC is capable of inducing the apoptosis of the target cells is the most critical measure of successful protein delivery for this particular target. Indeed, CYC (unlabeled, 1 µM) delivered by IBC-Pr had dose-dependent cytotoxic effects on HeLa cells, with an *IC*_50_ value of 7.5 μM for triggering apoptosis after 48 h (Fig. 5*A*). In contrast, free CYC displayed no cytotoxicity, even when the concentration was increased from 1 to 16 μM, as expected for a membrane-impermeable protein. The results demonstrated that IBC-Pr is not only capable of mediating effective cell internalization of CYC but also maintains the bioactivity of the protein throughout the course of transmembrane delivery. Therefore, IBC-Pr acts as a molecular membrane carrier for an active and fully functional protein. The retained activity is due to the reversibility of the supramolecular equilibria (see ITC results above); accordingly, while the binding of IBC-Pr is required to shuttle CYC through the membrane, it is sufficiently dynamic to allow its release inside the cytosol. Upon release, the apoptotic signaling cascade is initiated through tight, salt-bridged binding of CYC by apoptotic protease activating factor 1 (Apaf-1) (118).

The Annexin V-FITC/PI apoptosis kit was employed to further characterize the activity of internalized CYC. As shown in Fig. 5*B*, weak green fluorescence owing to Annexin V-FITC was observed in HeLa cells treated with CYC (1 μM) in the absence of carrier. In contrast, in the presence of IBC-Pr (100 μM), the apoptotic cells exhibited blebbing of the Annexin V-FITC-stained cellular membrane and for the late apoptotic cells staining by PI was observed. The apoptotic activities of the different CYC samples were quantified according to the sum of the fraction of early apoptotic cells (Q3) to late apoptotic cells after 24 h (Q2, Fig. 5*C*). In the absence of IBC-Pr, no obvious signs of apoptosis induction were observed while in the presence of IBC-Pr, early apoptosis amounted to 19.9% and late apoptosis to 5.1%, summing up to 25.0%. This apoptotic effect is comparable to or greater than that observed with microheterogeneous formulations developed for CYC delivery, e.g., those using polyphenol-based (93), calcium carbonate-mineralized (94), or mesoporous silica (95) nanoparticles, at comparable loads of CYC (12 µg mL^–1^ *versus* 5 to 50 µg mL^–1^) as well as incubation times (24 h *versus* 6 to 48 h) (93–95). IBC-Pr, as a highly water-soluble supramolecular additive, bypasses the need for previous loading with CYC and can be homogeneously administered. We accordingly classify protein delivery via molecular boron carriers as highly efficient and practically advantageous.

### Membrane Translocation of CYC Occurs by Molecular Carrier-assisted Direct Permeation.

**Fig. 6.**
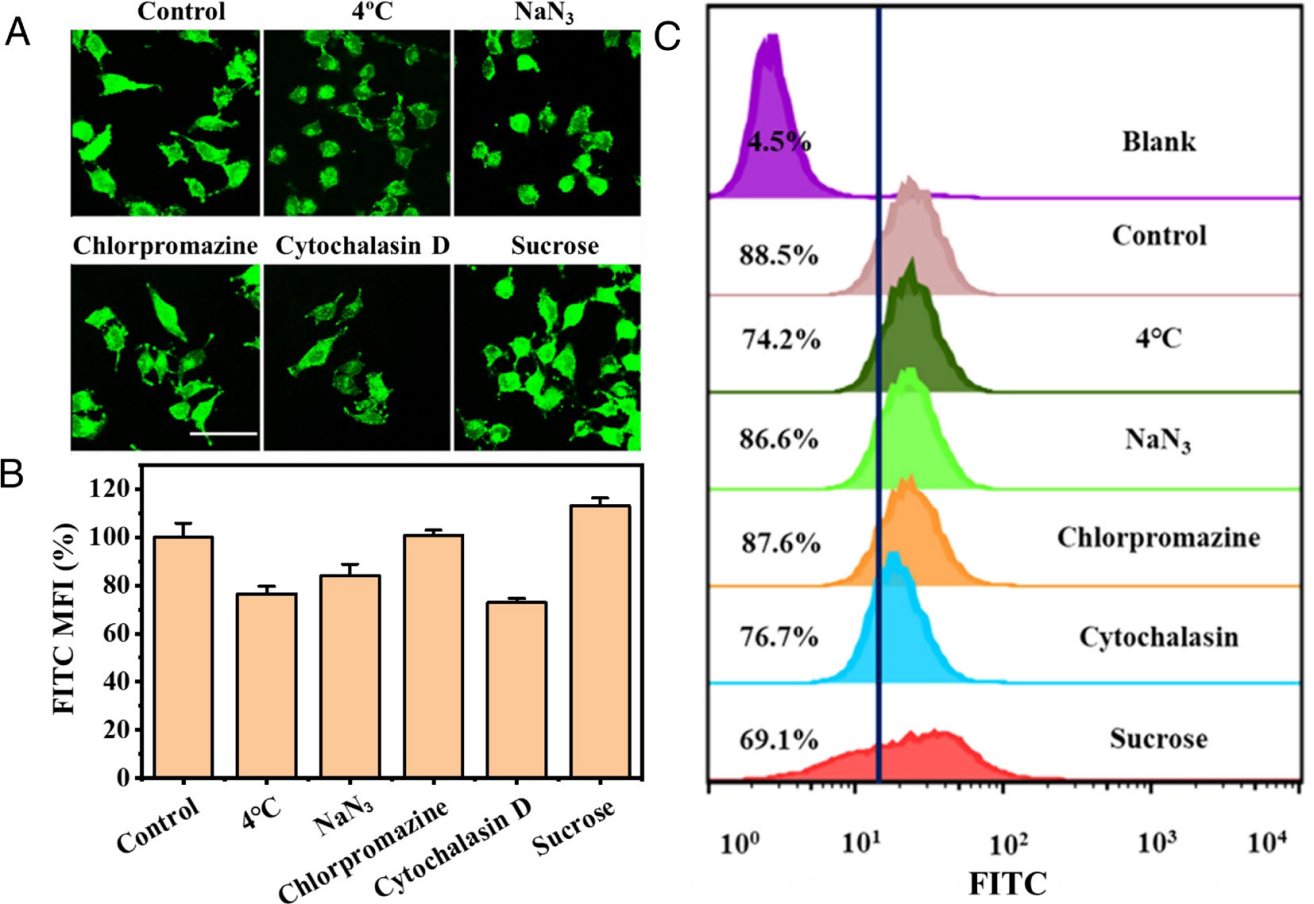
Membrane translocation by molecular carrier-assisted direct permeation. Cellular uptake efficiency of CYC (1 μM) delivered by IBC-Pr (100 μM) in the presence of different endocytosis inhibitors. (*A*) Confocal microscopy images, (*B*) relative mean fluorescence intensity per cell calculated from confocal microscopy images, and (*C*) quantitative intracellular fluorescence measured by flow cytometry (the threshold line was used to distinguish the FITC intensity of cells from the autofluorescence of blank cells). Representative images of three biological replicates are shown. Error bars indicate SD (*n* = 3).

Membrane translocation can occur through energy-dependent uptake pathways (prominently endocytosis), while the main energy-independent internalization pathway is direct permeation (119, 120). In principle, the formation of pores caused by a carrier presents another energy-independent pathway, but as no leakage is observed in the CF assays with large unilamellar vesicles (up to 1.2 mM, *SI Appendix*, Figs. S4 and S5), we do not consider this pathway for IBC-Pr. Instead, direct permeation through the lipid bilayer vesicles applies, and this was visually corroborated by observing the boron-cluster-mediated uptake of the fluorescently labeled protein (CYC-FITC) into giant vesicles (Fig. 3*D*). To determine the mechanism underlying the cellular uptake of the CYC/IBC-Pr system, confocal microscopic imaging (Fig. 6 *A* and *B*) and flow cytometry (Fig. 6*C*) were performed to evaluate the intracellular delivery efficiency under different pretreatment conditions. Internalization of CYC-FITC was monitored by its relative intracellular fluorescence intensity. At 4 °C, the fluorescence intensity was only slightly reduced, which ruled out a predominant energy-dependent uptake mechanism (121) as a predominant pathway. When cells were pretreated with NaN_3_, which causes ATP depletion, the relative mean fluorescence intensity decreased only slightly, which also suggested a dominant energy-independent internalization mechanism (122). Finally, two clathrin-dependent endocytic inhibitors, chlorpromazine and sucrose, as well as an actin-dependent endocytic inhibitor, cytochalasin D, were used to pretreat the cells. The experimental results showed that the uptake of CYC-FITC delivered by IBC-Pr was not strongly affected by these conventional inhibitors, and a high delivery capacity (70% or greater) remained. These findings, along with the homogeneous distribution (fluorescence) of CYC-FITC within the cells, corroborate an “energy-independent” uptake mechanism, namely, lipid bilayer permeation, as a dominant pathway. This direct permeation pathway is the same that is operative in the vesicle experiments, where energy-dependent pathways are not involved. The major circumvention of endocytic energy-dependent uptake routes (which are competitive but functionally unproductive) markedly improves the actual protein delivery efficiency into the cytosol; this accounts for the excellent bioactivity of CYC when it is delivered with the boron cluster. The molecular cluster carrier therefore stands out in terms of qualitative (no formulation, no loading, direct cytosolic transport) performance indicators.

## Discussion

Perhalogenated boron cluster anions have recently been shown to function as broadband molecular carriers (60, 61). The uptake mechanism has been related to the superchaotropic nature of these inorganic anions and the chaotropic effect, which enables dynamically reversible, supramolecular, and simultaneous interactions of the cluster anions with both the cargo and the lipid bilayer membrane (60, 89). By using a propyloxy derivative as a molecular carrier and cytochrome c (CYC) as cargo, we have shown that the method can be used to transport a real protein target, which broadens the application scope considerably, in the direction of functional cell biology and, potentially, therapy. Most importantly, the structural integrity as well as biological activity of the protein are retained both in vitro and in live cells, affording pronounced biological effects (apoptosis) at low cargo and carrier loads and implying highly efficient uptake. The uptake mechanism proceeds through the preferred route of direct bilayer permeation, which largely bypasses complications due to endocytic uptake. The use of superchaotropic molecular carriers, namely, boron cluster anions, offers numerous advantages because no microheterogeneous formulations are required, cargo and carrier can be added in situ, sequentially or together, and their relative concentrations can be adjusted at will and, beforehand, optimized for vesicles as model membranes. Moreover, the small size, low molecular weight, and high water solubility of the clusters are attractive from an administration and biocompatibility point of view. This method, which is highly complementary to the use of nanocarriers, should be transferable to other proteins, prominently positively charged ones, which have been notoriously difficult to target. Also conceivable, monofunctionalization of perbrominated boron clusters (97, 98) with substituents other than propyloxy offers perspectives for a direct attachment to the deliverable cargo or to targeting groups to achieve selective cellular uptake. While therapeutic applications may appear more far-fetched, the recently introduced method can be conveniently used in cell biology for studying protein uptake and subsequent activity in the cytosol.

## Methods

Na_2_B_12_Br_11_OCH_2_CH_2_CH_3_ (IBC-Pr) was synthesized as previously reported (97, 99). Circular dichroism (CD) spectra were recorded on a MOS-450/AF-CD instrument (France) and the ratio of secondary structures was calculated by software K2D2 (123). The enzyme-catalytic activity of CYC was measured through the conversion of 2,2’-azino-bis(3-ethylbenzthiazoline-6-sulfonate) (ABTS) to its radical cation, ABTS^⋅+^ (124). Phospholipid concentrations of the prepared vesicles were determined by the Stewart assay (125). Transport experiments in vesicles were performed with the carboxyfluorescein (CF) assay. Giant vesicles were formed by electroformation (56, 126, 127). Labeling of CYC with FITC and TAMRA was performed by standard protocols. The cells for the uptake experiments, flow cytometry, and the cytotoxicity assays were cultured in Dulbecco´s modified eagle´s medium (DMEM) supplemented with 10% (v/v) fetal bovine serum (FBS) in a humidified 5% CO_2_ atmosphere inside an incubator at 37 °C. The cells used for all the experiments were in the logarithmic phases of growth. The Annexin V-FITC/PI kit was used to evaluate the ability of the CYC/IBC-Pr mixture to induce apoptosis. Additional details are provided in *SI Appendix*.

## Supplementary Material

Appendix 01 (PDF)

## Data Availability

All study data are included in the article and/or *SI Appendix*.

## References

[1] Y. Zhang, J. J. Roise, K. Lee, J. Li, N. Murthy, Recent developments in intracellular protein delivery. Curr. Opin. Biotechnol. **52**, 25–31 (2018). 29486392 10.1016/j.copbio.2018.02.009 PMC6082692

[2] B. Leader, Q. J. Baca, D. E. Golan, Protein therapeutics: A summary and pharmacological classification. Nat. Rev. Drug Discovery **7**, 21–39 (2008). 18097458 10.1038/nrd2399

[3] S. Du, S. S. Liew, L. Li, S. Q. Yao, Bypassing endocytosis: Direct cytosolic delivery of proteins. J. Am. Chem. Soc. **140**, 15986–15996 (2018). 30384589 10.1021/jacs.8b06584

[4] Y. W. Lee , Protein delivery into the cell cytosol using non-viral nanocarriers. Theranostics **9**, 3280–3292 (2019). 31244954 10.7150/thno.34412 PMC6567963

[5] W. Zhou, H. Cui, L. Ying, X. F. Yu, Enhanced cytosolic delivery and release of CRISPR/Cas9 by black phosphorus nanosheets for genome editing. Angew. Chem. Int. Ed. **130**, 10268–10272 (2018). 10.1002/anie.201806941 29939484

[6] R. Mout , General strategy for direct cytosolic protein delivery via protein-nanoparticle co-engineering. ACS Nano **11**, 6416–6421 (2017). 28614657 10.1021/acsnano.7b02884 PMC5766003

[7] A. Dinca, W. M. Chien, M. T. Chin, Intracellular delivery of proteins with cell-penetrating peptides for therapeutic uses in human disease. Int. J. Mol. Sci. **17**, 263 (2016). 26907261 10.3390/ijms17020263 PMC4783992

[8] J. A. Zuris , Cationic lipid-mediated delivery of proteins enables efficient protein-based genome editing in vitro and in vivo. Nat. Biotechnol. **33**, 73–80 (2015). 25357182 10.1038/nbt.3081 PMC4289409

[9] T. A. Slastnikova, A. V. Ulasov, A. A. Rosenkranz, A. S. Sobolev, Targeted intracellular delivery of antibodies: The state of the art. Front. Pharmacol. **9**, 1208 (2018). 30405420 10.3389/fphar.2018.01208 PMC6207587

[10] M. P. Deonarain, Q. Xue, Tackling solid tumour therapy with small-format drug conjugates. Antib. Ther. **3**, 237–245 (2020). 33928231 10.1093/abt/tbaa024 PMC7990258

[11] O. Dagher, T. R. King, N. Wellhausen, A. D. Posey Jr., Combination therapy for solid tumors: Taking a classic CAR on new adventures. Cancer Cell **38**, 621–623 (2020). 33064993 10.1016/j.ccell.2020.10.003

[12] Z. Gu, A. Biswas, M. Zhao, Y. Tang, Tailoring nanocarriers for intracellular protein delivery. Chem. Soc. Rev. **40**, 3638–3655 (2011). 21566806 10.1039/c0cs00227e

[13] V. J. Bruce, B. R. McNaughton, Inside job: Methods for delivering proteins to the interior of mammalian cells. Cell Chem. Biol. **24**, 924–934 (2017). 28781125 10.1016/j.chembiol.2017.06.014

[14] D. Morshedi Rad , A comprehensive review on intracellular delivery. Adv. Mater **33**, 2005363 (2021). 10.1002/adma.202005363 33594744

[15] J. Kreitz , Programmable protein delivery with a bacterial contractile injection system. Nature **616**, 357–364 (2023). 36991127 10.1038/s41586-023-05870-7 PMC10097599

[16] F. Jiang , N-terminal signal peptides facilitate the engineering of PVC complex as a potent protein delivery system. Sci. Adv. **8**, eabm2343 (2022). 35486720 10.1126/sciadv.abm2343 PMC9054023

[17] I. Vlisidou , The Photorhabdus asymbiotica virulence cassettes deliver protein effectors directly into target eukaryotic cells. eLife **8**, e46259 (2019). 31526474 10.7554/eLife.46259 PMC6748792

[18] G. L. Szeto , Microfluidic squeezing for intracellular antigen loading in polyclonal B-cells as cellular vaccines. Sci. Rep. **5**, 10276 (2015). 25999171 10.1038/srep10276 PMC4441198

[19] A. Sharei , A vector-free microfluidic platform for intracellular delivery. Proc. Natl. Acad. Sci. U.S.A. **110**, 2082–2087 (2013). 23341631 10.1073/pnas.1218705110 PMC3568376

[20] N. Pathak , Cellular delivery of large functional proteins and protein-nucleic acid constructs via localized electroporation. Nano Lett. **23**, 3653–3660 (2023). 36848135 10.1021/acs.nanolett.2c04374 PMC10433461

[21] C. A. Patino , Multiplexed high-throughput localized electroporation workflow with deep learning based analysis for cell engineering. Sci. Adv. **8**, eabn7637 (2022). 35867793 10.1126/sciadv.abn7637 PMC9307252

[22] S.-O. Choi , Intracellular protein delivery and gene transfection by electroporation using a microneedle electrode array. Small **8**, 1081–1091 (2012). 22328093 10.1002/smll.201101747 PMC3516926

[23] A. Fu, R. Tang, J. Hardie, M. E. Farkas, V. M. Rotello, Promises and pitfalls of intracellular delivery of proteins. Bioconjugate Chem. **25**, 1602–1608 (2014). 10.1021/bc500320j PMC4166028 25133522

[24] A. F. L. Schneider, M. Kithil, M. C. Cardoso, M. Lehmann, C. P. R. Hackenberger, Cellular uptake of large biomolecules enabled by cell-surface-reactive cell-penetrating peptide additives. Nat. Chem. **13**, 530–539 (2021). 33859390 10.1038/s41557-021-00661-x

[25] P. Säälik , Protein cargo delivery properties of cell-penetrating peptides. A comparative study. Bioconjugate Chem. **15**, 1246–1253 (2004). 10.1021/bc049938y 15546190

[26] R. Brock, The uptake of arginine-rich cell-penetrating peptides: Putting the puzzle together. Bioconjugate Chem. **25**, 863–868 (2014). 10.1021/bc500017t 24679171

[27] B. Zhitomirsky, Y. G. Assaraf, Lysosomal accumulation of anticancer drugs triggers lysosomal exocytosis. Oncotarget **8**, 45117–45132 (2017). 28187461 10.18632/oncotarget.15155 PMC5542171

[28] M. P. Stewart , In vitro and ex vivo strategies for intracellular delivery. Nature **538**, 183–192 (2016). 27734871 10.1038/nature19764

[29] R. Goswami, T. Jeon, H. Nagaraj, S. Zhai, V. M. Rotello, Accessing intracellular targets through nanocarrier-mediated cytosolic protein delivery. Trends Pharmacol. Sci. **41**, 743–754 (2020). 32891429 10.1016/j.tips.2020.08.005 PMC7502523

[30] S. Yu , Efficient intracellular delivery of proteins by a multifunctional chimaeric peptide in vitro and in vivo. Nat. Commun. **12**, 5131 (2021). 34446736 10.1038/s41467-021-25448-z PMC8390694

[31] N. Tamemoto , Rational design principles of attenuated cationic lytic peptides for intracellular delivery of biomacromolecules. Mol. Pharm. **17**, 2175–2185 (2020). 32352304 10.1021/acs.molpharmaceut.0c00312

[32] M. Ray, Y. W. Lee, F. Scaletti, R. Yu, V. M. Rotello, Intracellular delivery of proteins by nanocarriers. Nanomedicine (Lond) **12**, 941–952 (2017). 28338410 10.2217/nnm-2016-0393 PMC5829369

[33] E. Soprano, E. Polo, B. Pelaz, P. del Pino, Biomimetic cell-derived nanocarriers in cancer research. J. Nanobiotechnol. **20**, 538 (2022). 10.1186/s12951-022-01748-4 PMC9771790 36544135

[34] S. Le Saux , Interest of extracellular vesicles in regards to lipid nanoparticle based systems for intracellular protein delivery. Adv. Drug Deliv. Rev. **176**, 113837 (2021). 34144089 10.1016/j.addr.2021.113837

[35] M. Sakono, R. Hayakawa, Repressor-like on-off regulation of protein expression by the DNA-binding transcription activator-like effector in T7 promoter-based cell-free protein synthesis. ChemBioChem **22**, 888–893 (2021). 33085169 10.1002/cbic.202000591

[36] S. Le Saux , Nanotechnologies for intracellular protein delivery: Recent progress in inorganic and organic nanocarriers. Adv. Ther. **4**, 2100009 (2021).

[37] C. Chau, P. Actis, E. Hewitt, Methods for protein delivery into cells: From current approaches to future perspectives. Biochem. Soc. Trans. **48**, 357–365 (2020). 32267469 10.1042/BST20190039

[38] A. Hubbell Jeffrey, A. Chilkoti, Nanomaterials for drug delivery. Science **337**, 303–305 (2012). 22822138 10.1126/science.1219657

[39] Y. B. Kim, K. T. Zhao, D. B. Thompson, D. R. Liu, An anionic human protein mediates cationic liposome delivery of genome editing proteins into mammalian cells. Nat. Commun. **10**, 2905 (2019). 31266953 10.1038/s41467-019-10828-3 PMC6606574

[40] S. Banskota , Engineered virus-like particles for efficient in vivo delivery of therapeutic proteins. Cell **185**, 250–265 (2022). 35021064 10.1016/j.cell.2021.12.021 PMC8809250

[41] F. Scaletti , Protein delivery into cells using inorganic nanoparticle-protein supramolecular assemblies. Chem. Soc. Rev. **47**, 3421–3432 (2018). 29537040 10.1039/c8cs00008e PMC5962404

[42] J. Lv, Q. Fan, H. Wang, Y. Cheng, Polymers for cytosolic protein delivery. Biomaterials **218**, 119358 (2019). 31349095 10.1016/j.biomaterials.2019.119358

[43] M. Yan , A novel intracellular protein delivery platform based on single-protein nanocapsules. Nat. Nanotechnol. **5**, 48–53 (2010). 19935648 10.1038/nnano.2009.341

[44] Y. Sun , Phase-separating peptides for direct cytosolic delivery and redox-activated release of macromolecular therapeutics. Nat. Chem. **14**, 274–283 (2022). 35115657 10.1038/s41557-021-00854-4

[45] S. A. Smith, L. I. Selby, A. P. R. Johnston, G. K. Such, The endosomal escape of nanoparticles: Toward more efficient cellular delivery. Bioconjugate Chem. **30**, 263–272 (2019). 10.1021/acs.bioconjchem.8b00732 30452233

[46] J. López-Andarias , Cell-penetrating streptavidin: A general tool for bifunctional delivery with spatiotemporal control, mediated by transport systems such as adaptive benzopolysulfane networks. J. Am. Chem. Soc. **142**, 4784–4792 (2020). 32109058 10.1021/jacs.9b13621 PMC7307903

[47] C. Liu , Natural polyphenols augment cytosolic protein delivery by a functional polymer. Chem. Mater. **31**, 1956–1965 (2019).

[48] D. Argudo, N. P. Bethel, F. V. Marcoline, M. Grabe, Continuum descriptions of membranes and their interaction with proteins: Towards chemically accurate models. Biochim. Biophys. Acta **1858**, 1619–1634 (2016). 26853937 10.1016/j.bbamem.2016.02.003 PMC4877259

[49] S. Katayama , Effects of pyrenebutyrate on the translocation of arginine-rich cell-penetrating peptides through artificial membranes: Recruiting peptides to the membranes, dissipating liquid-ordered phases, and inducing curvature. Biochim. Biophys. Acta **1828**, 2134–2142 (2013). 23711826 10.1016/j.bbamem.2013.05.016

[50] T. Takeuchi , Direct and rapid cytosolic delivery using cell-penetrating peptides mediated by pyrenebutyrate. ACS Chem. Biol. **1**, 299–303 (2006). 17163758 10.1021/cb600127m

[51] M. Nishihara , Arginine magic with new counterions up the sleeve. Org. Biomol. Chem. **3**, 1659–1669 (2005). 15858647 10.1039/b501472g

[52] I. Pflueger , Cyclodextrin-based facial amphiphiles: Assessing the impact of the hydrophilic–lipophilic balance in the self-assembly, DNA complexation and gene delivery capabilities. Org. Biomol. Chem. **14**, 10037–10049 (2016). 27722597 10.1039/c6ob01882c

[53] C. O. Mellet, J. M. G. Fernández, J. M. Benito, Cyclodextrin-based gene delivery systems. Chem. Soc. Rev. **40**, 1586–1608 (2011). 21042619 10.1039/c0cs00019a

[54] Y. C. Pan , An amphiphilic sulfonatocalix[5]arene as an activator for membrane transport of lysine-rich peptides and proteins. Angew. Chem. Int. Ed. **60**, 1875–1882 (2021). 10.1002/anie.202011185 33051947

[55] D.-Y. Zhang , Structurally screening calixarenes as peptide transport activators. Chem. Commun. **57**, 12627–12630 (2021). 10.1039/d1cc05414g 34761762

[56] A. Barba-Bon , Fluorescence monitoring of peptide transport pathways into large and giant vesicles by supramolecular host-dye reporter pairs. J. Am. Chem. Soc. **141**, 20137–20145 (2019). 31739668 10.1021/jacs.9b09563

[57] S. Peng , Phosphorylation-responsive membrane transport of peptides. Angew. Chem. Int. Ed. **56**, 15742–15745 (2017). 10.1002/anie.201707979 29024239

[58] F. Perret , Anionic fullerenes, calixarenes, coronenes, and pyrenes as activators of oligo/polyarginines in model membranes and live cells. J. Am. Chem. Soc. **127**, 1114–1115 (2005). 15669846 10.1021/ja043633c

[59] G. Gasparini, E.-K. Bang, J. Montenegro, S. Matile, Cellular uptake: Lessons from supramolecular organic chemistry. Chem. Commun. **51**, 10389–10402 (2015). 10.1039/c5cc03472h 26030211

[60] A. Barba-Bon , Boron clusters as broadband membrane carriers. Nature **603**, 637–642 (2022). 35322251 10.1038/s41586-022-04413-w PMC8942850

[61] G. Salluce , Size and polarizability of boron cluster carriers modulate chaotropic membrane transport. Angew. Chem. Int. Ed. **63**, e202404286 (2024). 10.1002/anie.202404286 38712936

[62] Y. Chen , Metallacarborane cluster anions of the cobalt bisdicarbollide-type as chaotropic carriers for transmembrane and intracellular delivery of cationic peptides. J. Am. Chem. Soc. **145**, 13089–13098 (2023). 37265356 10.1021/jacs.3c01623 PMC10288510

[63] A. Barba-Bon , All-inorganic polyoxometalates act as superchaotropic membrane carriers. Adv. Mater **36**, 2309219 (2024). 37943506 10.1002/adma.202309219 PMC11475408

[64] J. L. Barton , Perfunctionalized dodecaborate clusters as stable metal-free active materials for charge storage. ACS Appl. Energy Mater. **2**, 4907–4913 (2019). 33778417 10.1021/acsaem.9b00610 PMC7996373

[65] J. Plesek, Potential applications of the boron cluster compounds. Chem. Rev. **92**, 269–278 (1992).

[66] E. L. Muetterties , Salts and acids of B10H10-2 and B1212–2. Inorg. Chem. **3**, 444–451 (1964).

[67] S. El Anwar , Versatile, one-pot introduction of nonahalogenated 2-ammonio-decaborate ions as boron cluster scaffolds into organic molecules; host-guest complexation with gamma-cyclodextrin. Chem. Commun. **55**, 13669–13672 (2019). 10.1039/c9cc07678f 31663544

[68] W. Wang , The chaotropic effect as an orthogonal assembly motif for multi-responsive dodecaborate-cucurbituril supramolecular networks. Chem. Commun. **54**, 2098–2101 (2018). 10.1039/c7cc08078f 29319071

[69] K. I. Assaf , Water structure recovery in chaotropic anion recognition: high-affinity binding of dodecaborate clusters to γ-cyclodextrin. Angew. Chem. Int. Ed. **54**, 6852–6856 (2015). 10.1002/anie.201412485 PMC4510780 25951349

[70] N. A. Bernier , Ex vivo and In vivo evaluation of dodecaborate-based clusters encapsulated in ferumoxytol nanoparticles. Langmuir **37**, 14500–14508 (2021). 34843246 10.1021/acs.langmuir.1c02506 PMC8761388

[71] E. Hey-Hawkins, C. V. Teixidor, Boron-based compounds: Potential and Emerging Applications in Medicine (John Wiley & Sons; Hoboken, NJ, USA, 2018).

[72] J. C. Axtell, L. M. A. Saleh, E. A. Qian, A. I. Wixtrom, A. M. Spokoyny, Synthesis and applications of perfunctionalized boron clusters. Inorg. Chem. **57**, 2333–2350 (2018). 29465227 10.1021/acs.inorgchem.7b02912 PMC5985200

[73] D. Gabel, Boron clusters in medicinal chemistry: Perspectives and problems. Pure Appl. Chem. **87**, 173–179 (2015).

[74] N. S. Hosmane, “Boron-based nanomaterials: Technologies and applications” in Boron Science (CRC Press, 2012), **vol. 21**, pp 489–514.

[75] I. B. Sivaev, V. V. Bregadze, Polyhedral boranes for medical applications: Current status and perspectives. Eur. J. Inorg. Chem. **2009**, 1433–1450 (2009).

[76] S. O. Oloo, K. M. Smith, M. d. G. H. Vicente, Multi-functional boron-delivery agents for boron neutron capture therapy of cancers. Cancers **15**, 3277 (2023). 37444386 10.3390/cancers15133277 PMC10340061

[77] J. Li , Designed boron-rich polymeric nanoparticles based on nano-ion pairing for boron delivery. Chem. Eur. J. **26**, 14283–14289 (2020). 32492217 10.1002/chem.202001699

[78] A. H. Soloway , The chemistry of neutron capture therapy. Chem. Rev. **98**, 2389–2390 (1998). 11848966 10.1021/cr980493e

[79] P. Dullinger, D. Horinek, Solvation of nanoions in aqueous solutions. J. Am. Chem. Soc. **145**, 24922–24930 (2023). 10.1021/jacs.3c09494 37909095

[80] K. I. Assaf, W. M. Nau, Large anion binding in water. Org. Biomol. Chem. **21**, 6636–6651 (2023). 37548417 10.1039/d3ob00975k

[81] Y. Hirai , Boron clusters alter the membrane permeability of dicationic fluorescent DNA-staining dyes. ACS Omega **8**, 35321–35327 (2023). 37779949 10.1021/acsomega.3c05156 PMC10536875

[82] W. Wei, Hofmeister effects shine in nanoscience. Adv. Sci. **10**, e2302057 (2023). 10.1002/advs.202302057 PMC10401134 37211703

[83] S. Yao , Hofmeister effect in the Keggin-type polyoxotungstate series. Inorg. Chem. Front. **8**, 12–25 (2021).

[84] M. Hohenschutz, I. Grillo, O. Diat, P. Bauduin, How nano-ions act like ionic surfactants. Angew. Chem. Int. Ed. **59**, 8084–8088 (2020). 10.1002/anie.201916193 32125752

[85] K. I. Assaf , High-affinity binding of metallacarborane cobalt bis (dicarbollide) anions to cyclodextrins and application to membrane translocation. J. Org. Chem. **84**, 11790–11798 (2019). 31274306 10.1021/acs.joc.9b01688

[86] T. Buchecker , Polyoxometalates in the hofmeister series. Chem. Commun. **54**, 1833–1836 (2018). 10.1039/c7cc09113c 29308490

[87] R. Fernandez-Alvarez, V. Dordovic, M. Uchman, P. Matejicek, Amphiphiles without head-and-tail design: Nanostructures based on the self-assembly of anionic boron cluster compounds. Langmuir **34**, 3541–3554 (2017). 29144761 10.1021/acs.langmuir.7b03306

[88] B. Naskar, O. Diat, V. R. Nardello-Rataj, P. Bauduin, Nanometer-size polyoxometalate anions adsorb strongly on neutral soft surfaces. J. Phys. Chem. C **119**, 20985–20992 (2015).

[89] K. I. Assaf, W. M. Nau, The chaotropic effect as an assembly motif in chemistry. Angew. Chem. Int. Ed. **57**, 13968–13981 (2018). 10.1002/anie.201804597 PMC6220808 29992706

[90] M. Hohenschutz, P. Bauduin, C. G. Lopez, B. Förster, W. Richtering, Superchaotropic nano-ion binding as a gelation motif in cellulose ether solutions. Angew. Chem. Int. Ed. **62**, e202210208 (2023). 10.1002/anie.202210208 PMC10107358 36346946

[91] D. Awad , Halogenated dodecaborate clusters as agents to trigger release of liposomal contents. Chempluschem **80**, 656–664 (2015). 31973437 10.1002/cplu.201402286

[92] P. Fan, S. Stolte, D. Gabel, Interaction of organic compounds and boron clusters with new silica matrices containing the phosphatidylcholine headgroup. Anal. Methods **6**, 3045–3055 (2014).

[93] Y. Han , Polyphenol-based nanoparticles for intracellular protein delivery via competing supramolecular interactions. ACS Nano **14**, 12972–12981 (2020). 32997490 10.1021/acsnano.0c04197

[94] A. N. Koo , Calcium carbonate mineralized nanoparticles as an intracellular transporter of cytochrome c for cancer therapy. Chem.-Asian J. **10**, 2380–2387 (2015). 26235642 10.1002/asia.201500630

[95] E. Choi, D. K. Lim, S. Kim, Hydrolytic surface erosion of mesoporous silica nanoparticles for efficient intracellular delivery of cytochrome c. J. Colloid Interface Sci. **560**, 416–425 (2020). 31679782 10.1016/j.jcis.2019.10.100

[96] S. K. Kim, M. B. Foote, L. Huang, The targeted intracellular delivery of cytochrome C protein to tumors using lipid-apolipoprotein nanoparticles. Biomaterials **33**, 3959–3966 (2012). 22365810 10.1016/j.biomaterials.2012.02.010 PMC3307951

[97] J. Zhang, D. Gabel, K. I. Assaf, W. M. Nau, A fluorescein-substituted perbrominated dodecaborate cluster as an anchor dye for large macrocyclic hosts and its application in indicator displacement assays. Org. Lett. **24**, 9184–9188 (2022). 36507622 10.1021/acs.orglett.2c03615

[98] K. Fink, K. Kobak, M. Kasztura, J. Boratyński, T. M. Goszczyński, Synthesis and biological activity of thymosin β4-anionic boron cluster conjugates. Bioconjugate Chem. **29**, 3509–3515 (2018). 10.1021/acs.bioconjchem.8b00646 30365887

[99] C. Jenne, C. Kirsch, Alkoxy substituted halogenated closo-dodecaborates as anions for ionic liquids. Dalton Trans. **44**, 13119–13124 (2015). 26107425 10.1039/c5dt01633a

[100] V. Geis, K. Guttsche, C. Knapp, H. Scherer, R. Uzun, Synthesis and characterization of synthetically useful salts of the weakly-coordinating dianion [B_12_Cl_12_]^2-^. Dalton Trans. **15**, 2687–2694 (2009). 10.1039/b821030f 19333492

[101] M. V. Kuperman , Effective binding of perhalogenated closo -borates to serum albumins revealed by spectroscopic and ITC studies. J. Mol. Struct. **1141**, 75–80 (2017).

[102] T. M. Goszczyński, K. Fink, K. Kowalski, Z. J. Leśnikowski, J. Boratyński, Interactions of boron clusters and their derivatives with serum albumin. Sci. Rep. **7**, 9800 (2017). 28852112 10.1038/s41598-017-10314-0 PMC5574927

[103] I. I. Slowing, B. G. Trewyn, V.S.-Y. Lin, Mesoporous silica nanoparticles for intracellular delivery of membrane-impermeable proteins. J. Am. Chem. Soc. **129**, 8845–8849 (2007). 17589996 10.1021/ja0719780

[104] L. Hannibal , Alternative conformations of cytochrome c: Structure, function, and detection. Biochemistry **55**, 407–428 (2016). 26720007 10.1021/acs.biochem.5b01385

[105] A. B. Ghisaidoobe, S. J. Chung, Intrinsic tryptophan fluorescence in the detection and analysis of proteins: A focus on Forster resonance energy transfer techniques. Int. J. Mol. Sci. **15**, 22518–22538 (2014). 25490136 10.3390/ijms151222518 PMC4284722

[106] J. Mohanty, W. M. Nau, Refractive index effects on the oscillator strength and radiative decay rate of 2, 3-diazabicyclo [2.2. 2] oct-2-ene. Photochem. Photobiol. Sci. **3**, 1026–1031 (2004). 15570390 10.1039/b412936a

[107] D. Toptygin, R. S. Savtchenko, N. D. Meadow, S. Roseman, L. Brand, Effect of the solvent refractive index on the excited-state lifetime of a single tryptophan residue in a protein. J. Phys. Chem. B **106**, 3724–3734 (2002).

[108] I. Aviram, The interaction of chaotropic anions with acid Ferricytochrome C. J. Biol. Chem. **248**, 1894–1896 (1973). 4347855

[109] Y. Chen , Insights into the enhanced catalytic activity of cytochrome c when encapsulated in a metal-organic framework. J. Am. Chem. Soc. **142**, 18576–18582 (2020). 33048545 10.1021/jacs.0c07870

[110] F. Le Guern, V. Mussard, A. Gaucher, M. Rottman, D. Prim, Fluorescein derivatives as fluorescent probes for ph monitoring along recent biological applications. Int. J. Mol. Sci. **21**, 9217 (2020). 33287208 10.3390/ijms21239217 PMC7729466

[111] J. Cebula, K. Fink, J. Boratyński, T. M. Goszczyński, Supramolecular chemistry of anionic boron clusters and its applications in biology. Coord. Chem. Rev. **477**, 214940 (2023).

[112] Y.-L.P. Ow, D. R. Green, Z. Hao, T. W. Mak, Cytochrome C: Functions beyond respiration. Nat. Rev. Mol. Cell Biol. **9**, 532–542 (2008). 18568041 10.1038/nrm2434

[113] S. H. Hristova, A. M. Zhivkov, Isoelectric point of free and adsorbed cytochrome c determined by various methods. Colloids Surf. B Biointerfaces **174**, 87–94 (2019). 30445254 10.1016/j.colsurfb.2018.10.080

[114] S. Gurunathan , Enhanced green fluorescent protein-mediated synthesis of biocompatible graphene. J. Nanobiotechnology **12**, 41 (2014). 25273520 10.1186/s12951-014-0041-9 PMC4193993

[115] K. M. Kanal, G. D. Fullerton, I. L. Cameron, A study of the molecular sources of nonideal osmotic pressure of bovine serum albumin solutions as a function of pH. Biophys. J. **66**, 153–160 (1994). 8130335 10.1016/S0006-3495(94)80773-8 PMC1275675

[116] P. Ghosh , Intracellular delivery of a membrane-impermeable enzyme in active form using functionalized gold nanoparticles. J. Am. Chem. Soc. **132**, 2642–2645 (2010). 20131834 10.1021/ja907887z PMC2830715

[117] M. Morales-Cruz , Activation of caspase-dependent apoptosis by intracellular delivery of Cytochrome C-based nanoparticles. J. Nanobiotechnology **12**, 33 (2014). 25179308 10.1186/s12951-014-0033-9 PMC4237869

[118] D. N. Shalaeva, D. V. Dibrova, M. Y. Galperin, A. Y. Mulkidjanian, Modeling of interaction between cytochrome c and the WD domains of Apaf-1: Bifurcated salt bridges underlying apoptosome assembly. Biol. Direct **10**, 29 (2015). 26014357 10.1186/s13062-015-0059-4 PMC4445527

[119] D. Manzanares, V. Cena, Endocytosis: The nanoparticle and submicron nanocompounds gateway into the cell. Pharmaceutics **12**, 371 (2020). 32316537 10.3390/pharmaceutics12040371 PMC7238190

[120] H. Nakamura, S. Watano, Direct permeation of nanoparticles across cell membrane: A review. Kona Powder Part. J. **35**, 2018011 (2018).

[121] R. Rezgui, K. Blumer, G. Yeoh-Tan, A. J. Trexler, M. Magzoub, Precise quantification of cellular uptake of cell-penetrating peptides using fluorescence-activated cell sorting and fluorescence correlation spectroscopy. Biochim. Biophys. Acta **1858**, 1499–1506 (2016). 27033412 10.1016/j.bbamem.2016.03.023

[122] Y. Jiang , The interplay of size and surface functionality on the cellular uptake of sub-10 nm gold nanoparticles. ACS Nano **9**, 9986–9993 (2015). 26435075 10.1021/acsnano.5b03521 PMC5848075

[123] C. Perez-Iratxeta, M. A. Andrade-Navarro, K2D2: Estimation of protein secondary structure from circular dichroism spectra. BMC Structural Biology **8**, 25 (2008). 18477405 10.1186/1472-6807-8-25 PMC2397409

[124] C. Guo , Gold nanoparticle-guarded large-pore mesoporous silica nanocomposites for delivery and controlled release of cytochrome c. J. Colloid Interface Sci. **589**, 34–44 (2021). 33444821 10.1016/j.jcis.2020.12.117

[125] J. C. M. Stewart, Colorimetric determination of phospholipids with ammonium ferrothiocyanate. Anal. Biochem. **104**, 10–14 (1980). 6892980 10.1016/0003-2697(80)90269-9

[126] B. Apellániz, J. L. Nieva, P. Schwille, A. J. García-Sáez, All-or-none versus graded: Single-vesicle analysis reveals lipid composition effects on membrane permeabilization. Biophys. J. **99**, 3619–3628 (2010). 21112286 10.1016/j.bpj.2010.09.027 PMC2998612

[127] Z. Boban, I. Mardešić, W. K. Subczynski, M. Raguz, Giant unilamellar vesicle electroformation: What to use, what to avoid, and how to quantify the results. Membranes (Basel) **11**, 860 (2021). 34832088 10.3390/membranes11110860 PMC8622294

