# Supplementary material for: A small-molecule carrier for the intracellular delivery of a membrane-impermeable protein with retained bioactivity
Source: Proc Natl Acad Sci U S A. 2024 Oct 22;121(44):e2407515121. doi: 10.1073/pnas.2407515121 (PMC11536097; doi:10.1073/pnas.2407515121)
Supplement: Supplementary file 1 — Appendix 01 (PDF) [file pnas.2407515121.sapp.pdf]

PNAS

Supporting Information for

A Small-Molecule Carrier for the Intracellular Delivery of a Membrane-Impermeable Protein with Retained Bioactivity

Xiqi Ma[1,3], Zhixiong Zhang[1,3], Andrea Barba-Bon[2], Dongxue Han[1], Zichun Qi[1], Baosheng Ge[1], Hua He[1], Fang Huang[1,*], Werner M. Nau[1,2,*], and Xiaojuan Wang[1,*]

[1] College of Chemical Engineering, China University of Petroleum (East China), Qingdao 266580, China.

[2] School of Science, Constructor University, Campus Ring 1, 28759 Bremen, Germany.

[3] These authors contributed equally.

*Email: fhuang@upc.edu.cn; *Email: wnau@constructor.university; *Email: xwang@upc.edu.cn

This PDF file includes:

Figures S1 to S12

Table S1

Methods

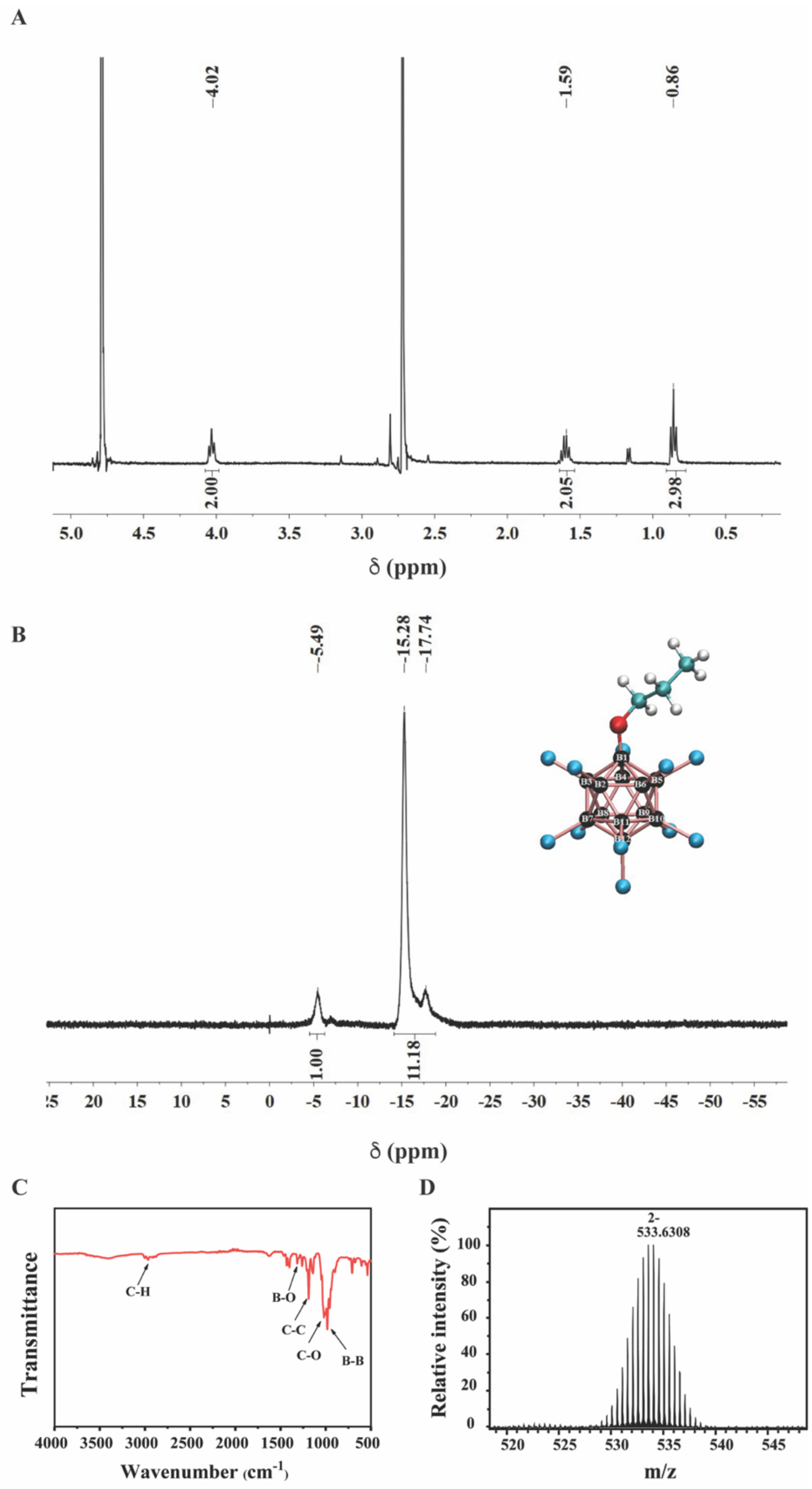

Fig. S1. (A) ^{1}H NMR spectrum (401 MHz) and (B) ^{11}B NMR spectrum (128 MHz) of $Na_2B_{12}Br_{11}OCH_2CH_2CH_3$ in D_2O at 298 K. (C) FTIR (diamond ATR) spectrum and (D) ESI-MS spectrum of $Na_2B_{12}Br_{11}OCH_2CH_2CH_3$. The ^{1}H NMR spectrum (A) showed a 2:2:3 integration pattern, which confirmed the presence of the propyloxy group of $Na_2B_{12}Br_{11}OCH_2CH_2CH_3$. The ^{11}B NMR spectrum (B) showed a 1:10:1 pattern. The oxygen-bonded boron B1 is observed at –5.49 ppm, and the antipodal boron atom B12 is at –17.74 ppm. The boron atoms B2-11 are at –15.28 ppm because the signals of the B2-6 and B7-11 atoms overlap. As shown in the FTIR spectrum (C), the B–B cage is observed at approximately 1000 cm^{-1}, and the B–Br vibrational peak is at 1021 cm^{-1}. The C–C vibrational peak is at 1188 cm^{-1} and the B–O peak is at 1314 cm^{-1}. The C–H vibration is observed at approximately 2900 cm^{-1}. The negative ESI-MS spectrum of $Na_2B_{12}Br_{11}OCH_2CH_2CH_3$ showed the complete anion as the main signal (D)^{11}B NMR (128 MHz, D_2O, 298 K) : δ = –5.49 (s, 1B, B1–O), –15.28 (s, 10B, B(2-11)–Br), δ = –17.74 (s, 1B, B12–Br); ^{1}H NMR (401 MHz, D_2O, 298 K): δ = 0.86 (3H, t, $^3J_{HH}$= 8.0 Hz, $[B_{12}Br_{11}OCH_2CH_2CH_3]^{2-}$), 1.59 (2H, m, $[B_{12}Br_{11}OCH_2CH_2CH_3]^{2-}$), 4.02 (2H, t, $^3J_{HH}$= 7.3 Hz, $[B_{12}Br_{11}OCH_2CH_2CH_3]^{2-}$). ESI-MS (negative mode, D_2O): *m*/*z*: 533.6308.

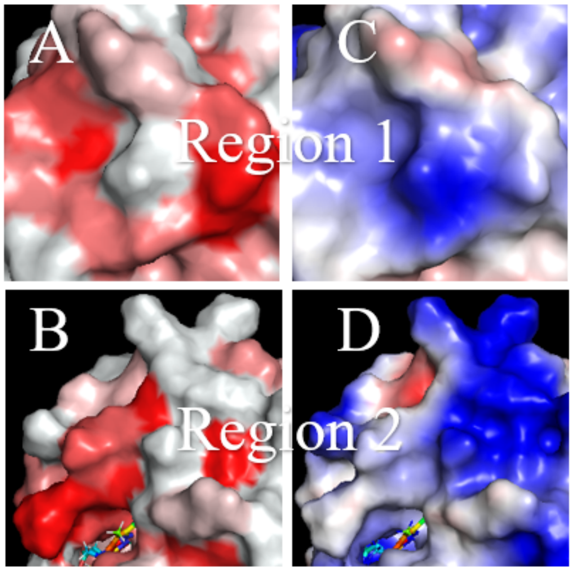

Fig. S2. (A) and (B) show the distributions of hydrophilicity and hydrophobicity on the protein surface in Region 1 and 2, respectively (red indicates hydrophobicity, gray indicates hydrophilicity); (C) and (D) show the surface charge distributions of proteins in Region 1 and 2, respectively (blue indicates positive and red negative charge density).

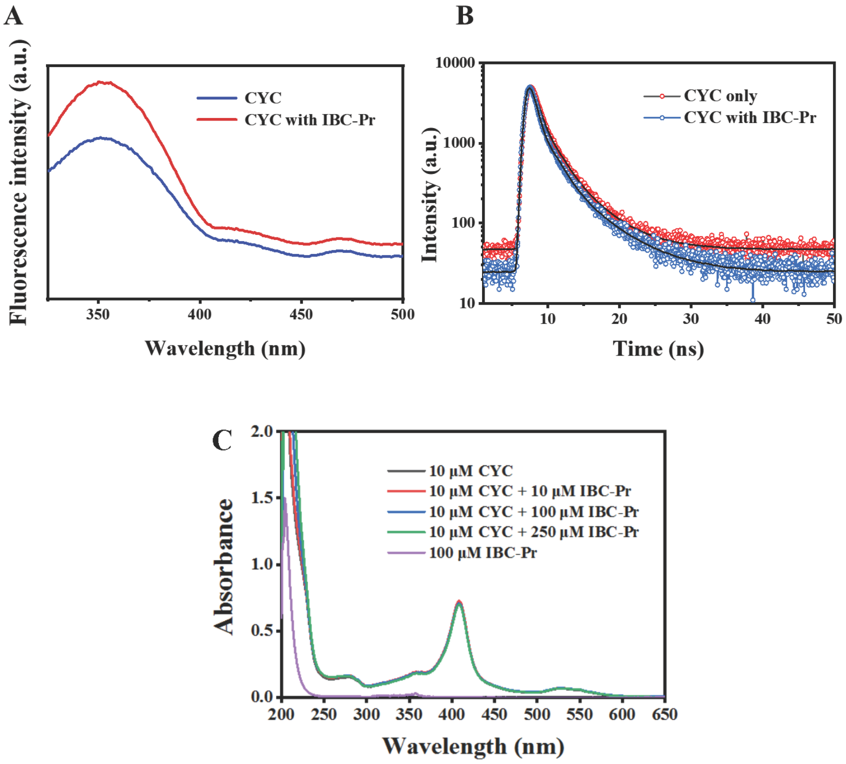

Fig. S3. (A) Fluorescence emission spectra and (B) time-resolved fluorescence decay traces of CYC (10 μM) in the absence and presence of IBC-Pr (20 μM) and (C) UV-Vis spectra of CYC (10 μM) in the absence and presence of IBC-Pr.

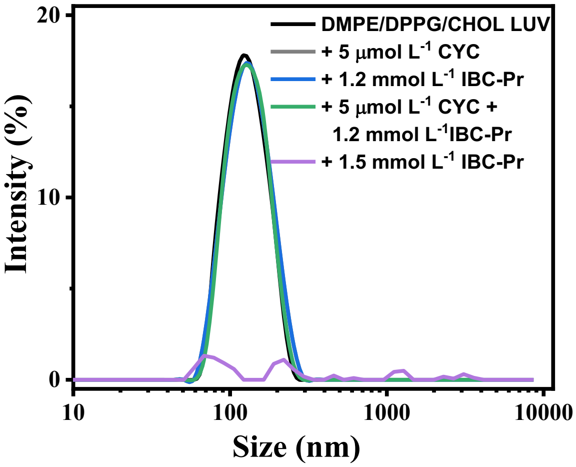

Fig. S4. Size distribution (by DLS) of DMPE/DPPG/CHOL (1/2/1) vesicles before and after addition of CYC (5 μM), IBC-Pr (1.2 mM and 1.5 mM), and the complex (5 μM CYC + 1.2 mM IBC-Pr), showing retention of membrane integrity up to 1.2 mM of boron cluster.

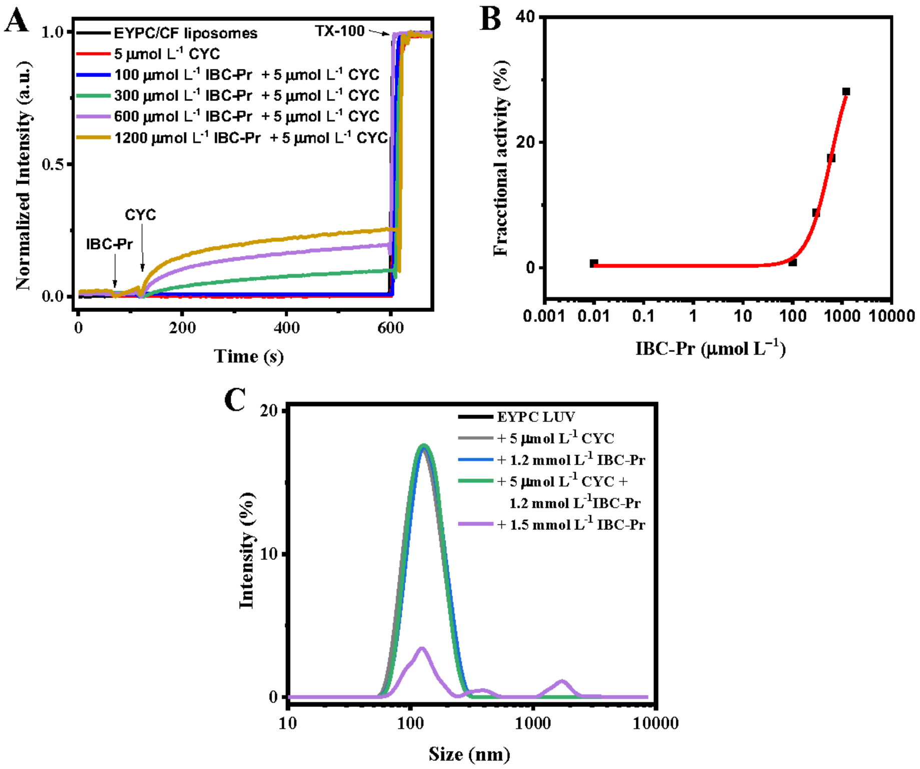

Fig. S5. (A) Changes in CF emission (l_{ex} = 492 nm, l_{em} = 517 nm) in CF-encapsulated EYPC liposomes as a function of time upon addition of different concentrations of IBC-Pr (0-1200 μM) at t = 60 s, CYC (5 μM) at t = 120 s, and TX-100 at t = 600 s, for calibration, and (B) fractional activity for IBC-Pr and the corresponding Hill curve fit. (C) Size distribution (by DLS) of EYPC vesicles before and after addition of CYC (5 μM), IBC-Pr (1.2 mM and 1.5 mM), and the complex (5 μM CYC + 1.2 mM IBC-Pr), showing the retention of membrane integrity up to 1.2 mM of cluster.

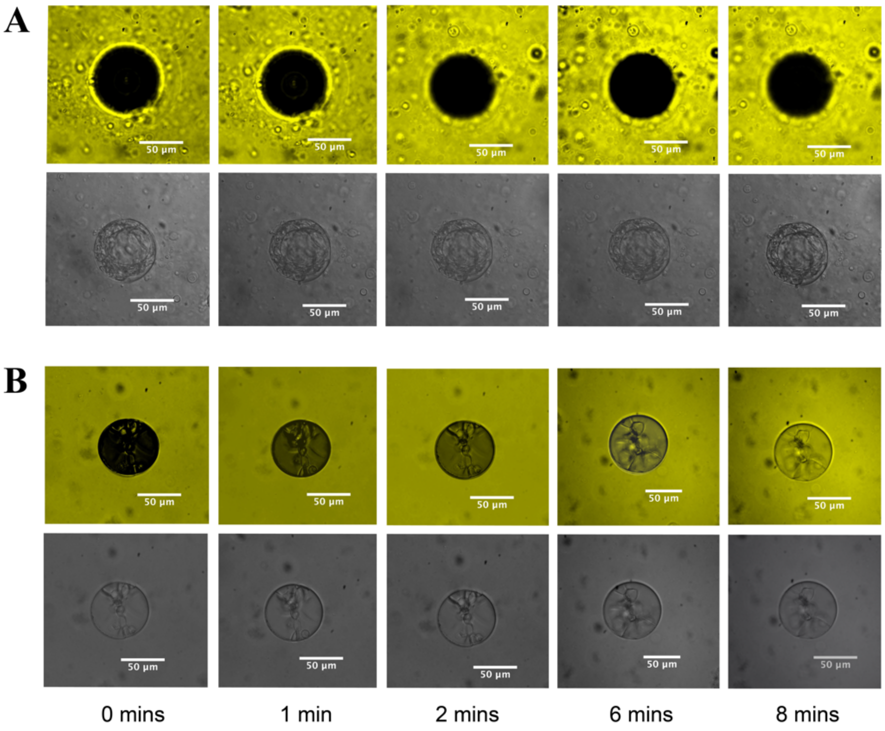

Fig. S6. Fluorescence (top rows) and bright-field (bottom rows) microscopy images of EYPC GUVs after the addition of CYC-FITC (10 μM) (A) in the absence and (B) in the presence of IBC-Pr (50 μM) recorded after different incubation times (0-8 min).

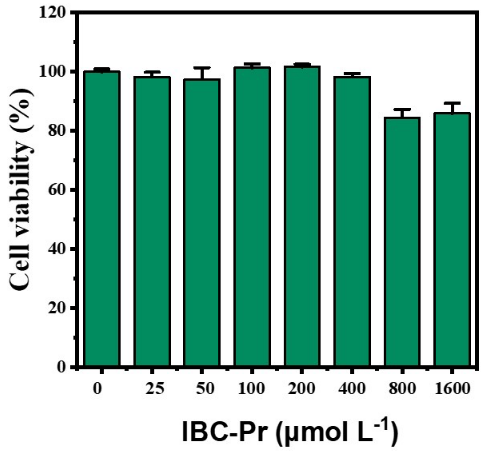

Fig. S7. Evaluation of the viability of HeLa cells treated with IBC-Pr as measured by MTT assay after 48 h of incubation. Error bars indicate SD (n = 3).

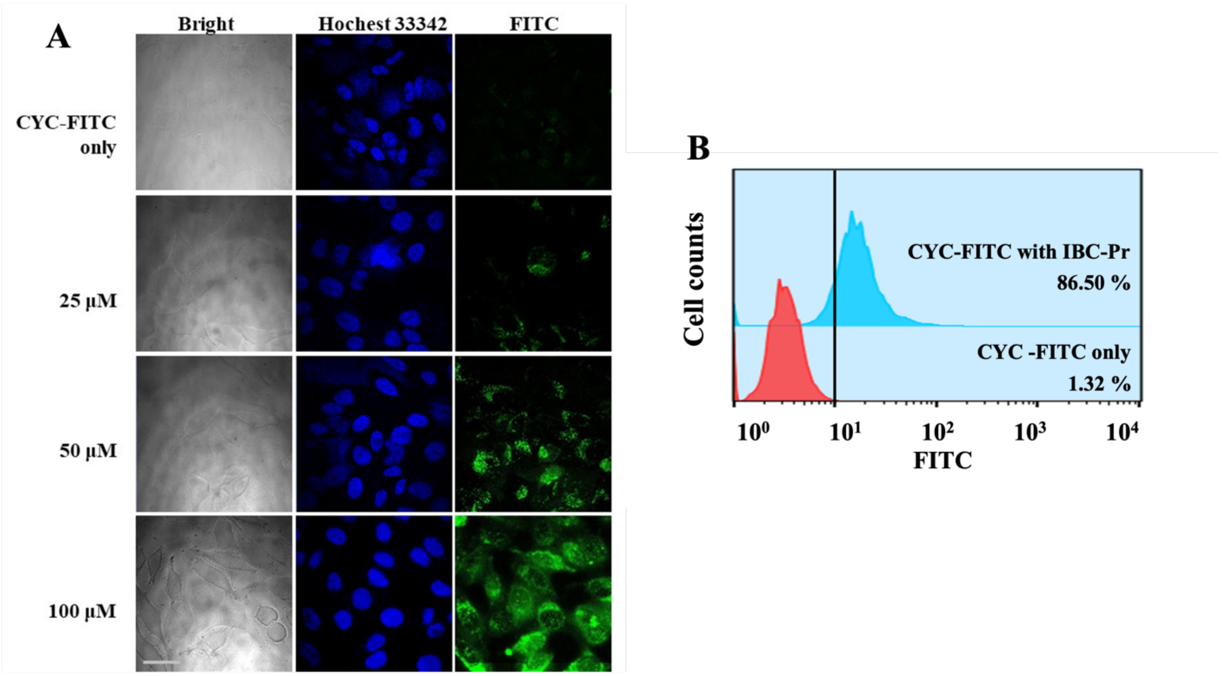

Fig. S8. (A) Live cell images of HeLa cells after incubation with CYC-FITC (1 μM) without or with IBC-Pr (0-100 μM) for 3 h. Representative images of three biological replicates. (B) Quantitative intracellular fluorescence as measured by flow cytometry after incubation of HeLa cells with CYC-FITC (1 μM) without or with IBC-Pr (100 μM).

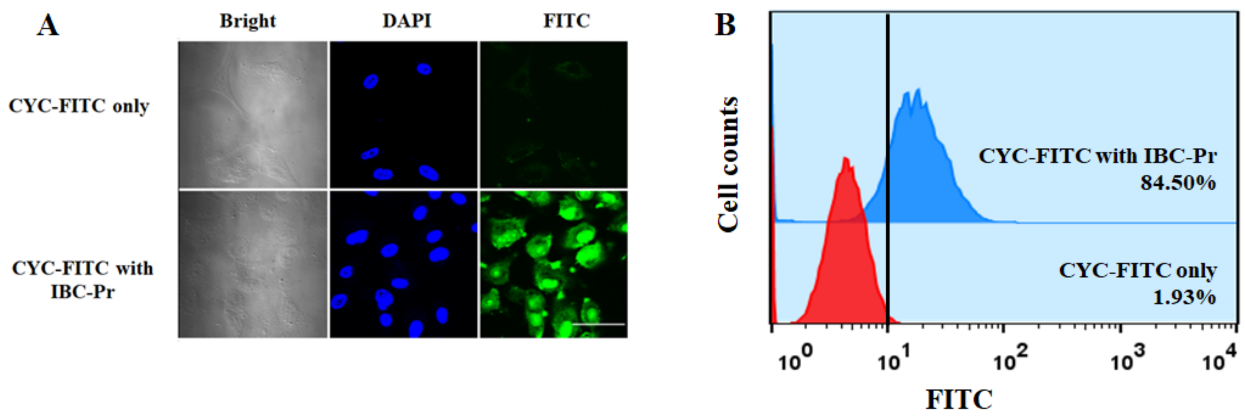

Fig. S9. (A) Confocal images of A549 cells after incubation with CYC-FITC (1 μM) without or with IBC-Pr (100 μM) for 3 h. Representative images of three biological replicates. (B) Quantitative intracellular fluorescence as measured by flow cytometry after incubation of HeLa cells with CYC-FITC (1 μM) without or with IBC-Pr (100 μM).

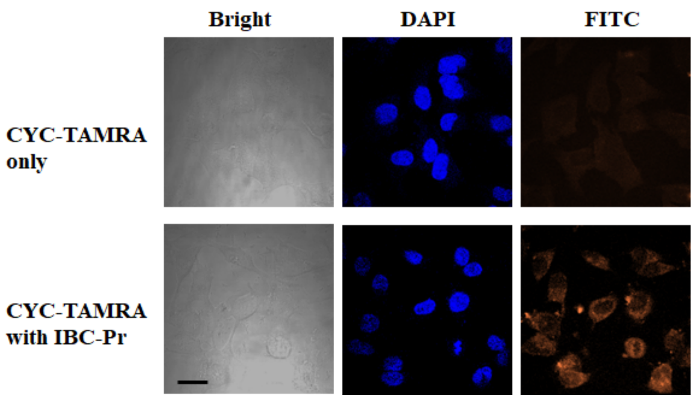

Fig. S10. Confocal images of HeLa cells after incubation with CYC-TAMRA (1 μM) without or with IBC-Pr (100 μM) for 3 h. Representative images of three biological replicates.

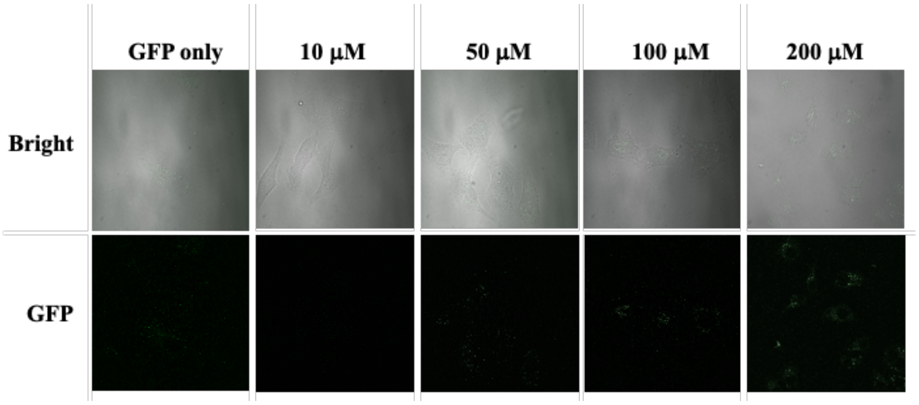

Fig. S11. Confocal images of HeLa cells after incubation with GFP (0.3 μM) without or with IBC-Pr for 3 h. Representative images of three biological replicates.

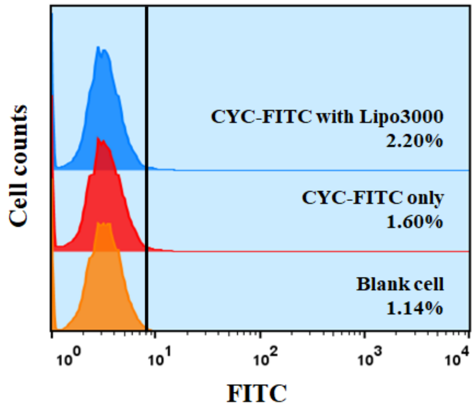

Fig. S12. Quantitative intracellular fluorescence as measured by flow cytometry after incubation of HeLa cells with CYC-FITC (1 μM) without or with Lipofectamine™ 3000 carrier; no significant uptake was observed.

Table S1. Cellular uptake of CYC-FITC (1μM) determined by flow cytometry.

Cell line	Carrier	Serum as additive	Uptake efficiency (%)[a]
HeLa	- - -	- - -	2.9 ± 2.1
	IBC-Pr[b]	- - -	87.7 ± 4.0
	Lipofectamine™ 3000[c]	- - -	3.0 ± 1.0
	- - -	10% (v/v) FBS	2.4 ± 0.9
	IBC-Pr[b]	10% (v/v) FBS	86.5 ± 4.5
A549	- - -	- - -	2.0 ± 1.2
	IBC-Pr[b]	- - -	84.5 ± 5.0

[a] Error refers to SD from triplicate measurements. [b] 100 μM. [c] According to manufacturer´s protocol in Opti-MEM medium.

Methods

Materials. All chemicals used for the synthesis of the boron clusters as well as H_2O_2, NaCl, KCl, Na_2HPO_4, and KH_2PO_4 were from Sinopharm Chemical Reagent Co., Ltd. (Shanghai, China). Lipofectamine™ 3000 was from Thermo Fisher Scientific Inc. (China). Reagents and compounds for buffer preparation and analytical measurements including carboxyfluorescein, 1,2-dimyristoyl-*sn*-glycero-3-phosphoethanolamine, 1,2-dipalmitoyl-*sn*-glycero-3-phospho-(1'-rac-glycerol), cholesterol, egg yolk phosphatidylcholine, HEPES, and sodium chloride were from Sigma-Aldrich (Germany). Triton X-100 (TX-100), Tris, and sucrose were from AppliChem (Germany). Cytochrome C (CYC), from equine heart, was from Glentham Life Sciences (UK) or from Yuanye Bio-Technology Co., Ltd. (Shanghai, China). The latter supplier was also used for 4’,6-diamidino-2-phenylindole (DAPI), and fluorescein isothiocyanate (FITC). Chloroform and methanol were from Carl Roth (Germany). 5-Carboxytetramethylrhodamin (TAMRA), Hoechst33342, propidium iodide (PI), 1-(3-dimethylaminopropyl)-3-ethylcarbodiimide (EDC) and N-hydroxy succinimide (NHS) were purchased from Macklin Biochemical Co., Ltd. (Shanghai, China). 3-(4,5-dimethylthiazol-2-yl)-2,5-diphenyltetrazolium bromide (MTT), fetal bovine serum (FBS), and Dulbecco's modified eagle medium (DMEM) were from Sangon Biotech Co., Ltd (Shanghai, China). Green fluorescent protein (GFP) was expressed in *E.coli* and purified by HPLC in house. The 2,2’-azino-bis(3-ethylbenzothiazoline-6-sulfonic acid) (ABTS) and Annexin V-FITC apoptosis detection kits were obtained from Beyotime Biotechnology (Shanghai, China). HeLa cells (human cervical carcinoma) and A549 cells (human lung adenocarcinoma cells) were obtained from Shanghai Institutes for Biology Sciences, Chinese Academy of Science.

Synthesis and characterization of IBC-Pr. $[B_{12}H_{12}OH]^{2-}$ was reacted with Br_2 to form $[B_{12}Br_{11}OH]^{2-}$, which is alkylated with 1-bromopropane to obtain the final product ($[B_{12}Br_{11}OCH_2CH_2CH_3]^{2-}$, IBC-Pr, as sodium salt), see ref. 97 and 99 in main text. FTIR spectra were obtained using a Nicolet Is5 spectrometer. NMR spectra were performed on a JEOL ECX 400 spectrometer and are

reported as chemical shifts (δ) in ppm relative to TMS (δ = 0) as the internal standard. ESI-HRMS spectra were recorded on a Bruker HCT Ultra spectrometer and are reported as the mass-to-charge ratio (m/z).

Isothermal titration calorimetry. ITC binding isotherms were recorded on a VP-ITC MicroCalorimeter from MicroCal, Int., at atmospheric pressure and 25°C. The solutions were degassed and thermostated prior to the titration experiments in a ThermoVac accessory. A constant volume of IBC-Pr (10 μL per injection) was injected into the CYC solution in water (250 μM) to determine the binding affinity of CYC for IBC-Pr. Dilution heats were determined by titration of IBC-Pr into water and subtracting the result from the reaction heat. The neat reaction heat was fitted with Origin 7.0 software by using a "*one-set-of-sites*" model to obtain the complex stability constant (K_a) and the molar interaction enthalpy (ΔH). The free energy (ΔG) and entropy changes (ΔS) were obtained according to $\Delta G = -RT\ln K_a = \Delta H - T\Delta S$.

Characterization of CYC in the absence and presence of IBC-Pr. CYC (1 μM) was mixed (with 100 μM IBC-Pr, where applicable) at room temperature in PBS (NaCl, 137.0 mM; KCl, 2.7 mM; Na_2HPO_4, 10.0 mM; KH_2PO_4, 2.0 mM; pH 7.4) for 20 min before the different sets of spectral measurements were taken. The size distributions and zeta potentials were measured on a Malvern Nano S instrument from Malvern Panalytical (USA). Fluorescence spectra of CYC without or with IBC-Pr were measured in a 1-cm quartz cuvette on a Hitachi F-2500 fluorescence spectrophotometer (Japan) at 25°C (λ_{exc} = 280 nm). Circular dichroism (CD) spectra of CYC (1 μM) were taken without and with IBC-Pr (100 μM) in PBS (pH 7.4). The CD spectra were recorded on a MOS-450/AF-CD instrument (France) by using a quartz cuvette with a 1-cm path length at a scan speed of 500 nm/min with a 0.5 s response and 0.1 nm interval; three scans were averaged. The final spectra were acquired by subtracting a blank spectrum containing only different concentrations of IBC-Pr and buffer. The software K2D2 (ref. 123 in main text) was used to estimate secondary structures. To measure the enzyme-catalytic activity of CYC, CYC (10 μM) and IBC-Pr (100 μM) were mixed for 20 minutes and the absorbance of the oxidized product ($ABTS^{\bullet+}$) at 418 nm was monitored via UV-Vis spectrophotometry (UV-1800 instrument, Japan), see ref. 124 in main text. The biocatalytic activity of native CYC was measured under the same conditions as a control.

Vesicle preparation. A thin lipid film was prepared by evaporating a solution of DMPE/DPPG/CHOL (4.4/10.4/2.6 mg, 1/2/1 molar ratio) in a 1:1 mixture of $CHCl_3$ and MeOH (1 mL) with a stream of nitrogen and, subsequently, under vacuum overnight. The dry film was hydrated at 55°C with 1 mL of buffer (100 mM CF, 10 mM Tris, pH 7.4) for 60 min and subjected to 10 freeze-thaw cycles and extrusions (15 times) through a polycarbonate membrane (pore size: 100 nm). Extravesicular components were removed by size exclusion chromatography (NAP-25 column Sephadex G-25 DNA grade) with 10 mM Tris and 140 mM NaCl, pH 7.4. The Stewart assay was used to determine lipid concentrations, see ref. 125 in main text. The EYPC vesicles were prepared analogously with the following modifications: 25 mg of EYPC in $CHCl_3$ (1 mL), and the film was hydrated at room temperature for 30 min with 50 mM CF in 10 mM HEPES (pH 7.5); size exclusion chromatography was performed with 10 mM HEPES and 107 mM NaCl (pH 7.5).

Transport experiments in vesicles. For the carboxyfluorescein (CF) assay, the DMPE/DPPG/CHOL⊃CF (or EYPC⊃CF) stock solution was diluted with buffer (10 mM Tris, 140 mM NaCl, pH 7.4 or 10 mM HEPES, 107 mM NaCl, pH 7.5) in a disposable plastic cuvette and gently stirred (total volume: 2 mL, final lipid concentration: 13 μM). CF fluorescence was monitored at 492 nm (λ_{exc} = 517 nm) as a function of time after addition of IBC-Pr (0-1200 μM) at 60 s, CYC (5 μM) at 120 s, and 24 μl of 1.2% (wt/vol) TX-100 at 600 s to lyse the vesicles, for calibration. Fluorescence intensities were normalized to fractional emission intensity as $I(t) = (I_t - I_0)/(I_\infty - I_0)$, where $I_0 = I_t$ before IBC-Pr addition and $I_\infty = I_t$ after lysis. For Hill analysis, I_f before lysis was defined as transmembrane activity, Y, and plotted against the IBC-Pr concentration, c, and fitted to the Hill equation, $Y = Y_0 + (Y_{max} - Y_0)/1+(EC_{50}/c)^n$, to afford the membrane activity in the absence of IBC-Pr, Y_0, the maximal membrane activity, Y_{max}, the concentration needed to achieve 50% maximal membrane activity, EC_{50}, and the Hill coefficient, n. It should be noted that CYC could not be transported through a bulk chloroform phase in U-tube experiments; this finding is in line with the lower transport efficiency of this larger protein (MW ca. 12 kDa, EC_{50} = 770 μM) compared to shorter cationic peptides (MW < 5 kDa, EC_{50} ca. 10 μM).(60)

Giant vesicle formation. Electro-formation (according to ref. 56,126,127 in main text) was performed at 3.0 V, 5 Hz, at 37 °C for 2 h in a Vesicle Prep Pro apparatus (Nanion, Germany). Briefly, 20 μL of EYPC solution (25 mg/mL in $CHCl_3$) were spread onto an ITO-coated glass slide. After solvent evaporation, the dried film was rehydrated with 300 mM sucrose solution and covered with another ITO slide.

Transport experiments with giant vesicles. Fluorescence microscopy of the giant vesicles was performed with an Axiovert 200 instrument (Carl Zeiss, filter set 44, i.e., BP 455-495 nm and LP 515 nm) equipped with a digital camera (Thorlabs Compact Scientific CS895MU) through a 20× objective on samples prepared by adding 10 μL of vesicle suspension on a glass slide and 40 μL of 300 mM sucrose. For the transport experiments, 50 μM of IBC-Pr and 10 μM of CYC-FITC were added to the GUV suspension and images were taken subsequently every minute. After 8 minutes, an aliquot of dilute HCl solution (pH 5.0) was added for acidification. To avoid possible photobleaching, samples were irradiated only when images were taken. The analysis of the images was performed in ImageJ software.

Labeling of CYC with FITC. CYC was labeled with FITC according to the manufacturer's standard protocol. Briefly, CYC (4 mg) was dissolved in 2 mL of 0.1 M sodium bicarbonate solution (pH 9.0). 500 μL of FITC (4 mg mL^{-1}) in DMSO was added to the CYC solution. The mixture was stirred at 4°C in the dark for 14 h, followed by addition of NH_4Cl to a final concentration of 50 mM, and then incubated for 2 hours at 4°C. The resulting solution was isolated by size exclusion chromatography with Sephadex G-25 as the stationary phase and PBS (pH 9.0) as the mobile phase. Finally, the CYC concentration and labeling efficiency (> 200%, that is, the molar ratio of FITC to CYC was more than 2:1) were measured via UV-Vis absorption spectroscopy. Note that the conformation of CYC (conformation by CD and DLS) did not significantly change upon FITC conjugation. The resulting CYC-FITC was stored at –20°C.

Labeling of CYC with TAMRA. TAMRA (8.76 mg) was dissolved in 5 mL of PBS buffer solution (NaCl, 137.0 mM; KCl, 2.7 mM; Na_2HPO_4, 10.0 mM; KH_2PO_4, 2.0 mM; pH 7.4). Subsequently, 1-(3-dimethylaminopropyl)-3-ethylcarbodiimide (7.8 mg) and N-hydroxy

succinimide (4.68 mg) were added, and the mixture was stirred at room temperature for 1 h. CYC (6 mg) was dissolved in 2 mL of PBS buffer solution, which was added dropwise to the TAMRA solution while stirring in the dark at 4°C for 12 h. Following this, the mixture was transferred to a dialysis bag with a molecular weight cutoff of 500D and purified via dialysis for 12 h.

Fixed-cell experiments to follow cellular uptake. Cells were seeded onto 12-mm sterile coverslips in a 24-well culture dish at a density of 1.0×10^5 cells per well overnight before protein delivery. After washing the cells thrice with PBS, the culture medium was replaced with fresh DMEM containing CYC-FITC (or CYC-TAMRA,1 μM each) and IBC-Pr (0-100 μM). After incubating for 3 h, the medium was discarded, and the cells were washed with PBS three times. Subsequently, the cells were fixed in 4% paraformaldehyde solution at ambient temperature and stained by the fluorescent dye DAPI. Subsequently, the side of the coverslip with the fixed cells was topped by a glass slide with 10 μL of 50% glycerol. Fluorescent images were taken by confocal laser scanning microscopy (CLSM, Nikon Al, Nikon). DAPI, FITC, and TAMRA were excited at 405, 488, and 532 nm, respectively, and their emission signals were collected between 425–475, 500–530, 552-617 nm, respectively. To evaluate a potential influence of serum on the uptake efficacy, fresh culture medium with 10% (v/v) FBS containing CYC-FITC and IBC-Pr was used to incubate with the cells. Experiments with GFP were conducted analogously, without DAPI staining; a 488 nm excitation laser was used, and the emission was collected between 500–530 nm.

Live-cell experiments to follow cellular uptake. About 1.0×10^4 cells were seeded into a glass-bottomed 8-well plate (200 μL/well) and grown until 90% confluence. After washing the cells thrice with PBS, the culture medium was replaced with fresh DMEM containing CYC-FITC (1 μM) and IBC-Pr (0-100 μM). After incubation for 3 h, the medium was discarded and washed with PBS. Subsequently, the cells were stained by the fluorescent dye Hoechst33342 and washed thrice with PBS. Fluorescence images were taken directly by confocal laser scanning microscopy (CLSM, Nikon Al, Nikon). Hoechst33342 was excited at 405 nm and FITC at 488 nm, and their emission signals were collected between 425-475 nm and 500-530 nm, respectively

Flow cytometry. Cells were seeded on plates at a density of 1.0×10^5 cells per well and cultured overnight. On the next day, the cells were washed thrice with PBS and incubated in fresh DMEM containing CYC-FITC (1 μM) and IBC-Pr (0-100 μM) for 3 h. After washing thrice with PBS, the cells were digested with trypsin until they were on the verge of detaching from the wells. Subsequently, PBS containing 20% serum was added to halt the digestion process. After centrifugation at 1000 rpm for 5 min, fresh PBS buffer was introduced and thoroughly mixed by pipetting. The delivery of CYC was assessed next, by using flow cytometry (FACSCalibur instrument, BD Bioscience, Franklin Lakes, NJ, USA). During detection, the cells were excited at 488 nm and the fluorescence signal between 510 - 540 nm was used to quantitatively analyze the transfection efficiency. The cell count for each detection was 1.0×10^4 cells and each sample was measured in triplicate. To assess the protein delivery efficiency of a commercially used carrier, Lipofectamine^{TM} 3000 in Opti-MEM medium containing CYC-FITC (1 μM) was prepared and incubated with the cells according to the protocol for flow cytometry.

Cytotoxicity assay. The 3-(4,5-dimethylthiazol-2-yl)-2,5-diphenyltetrazolium bromide (MTT) assay was used to determine cell viability. HeLa cells were seeded in 96-well plates at a density of 1.0×10^4 cells per well and subsequently incubated with fresh media containing IBC-Pr, free CYC, or CYC/IBC-Pr at various concentrations for 48 h. After cultivation, fresh media containing MTT reagents (0.5 mg mL^{-1}) was added to each well, and the cells were incubated for another 4 h at 37°C with 5% CO_2. Subsequently, the medium in each well was replaced with DMSO (100 μL) to dissolve the emerging formazan crystals. The absorbance at 490 nm of the samples was measured on a microplate reader (Molecular Devices Filter F5, China).

Apoptosis assay. The Annexin V-FITC/PI kit was used. Briefly, HeLa cells were incubated at a density of 1.0×10^5 cells per well in plain 24-well plates and incubated overnight. After 3 washes with PBS, DMEM medium (with 10% fetal bovine serum) containing CYC alone, IBC-Pr, or CYC/IBC-Pr (1 μM CYC and 100 μM IBC-Pr) was added for 24 h. The residual medium was aspirated, and the cells were incubated with PBS buffer containing 10% fetal bovine serum. The resulting medium was aspirated again, the cells were washed 3 times with PBS, and 195 μL of Annexin V-FITC binding buffer was added to each well. 5 μL of Annexin V-FITC staining solution was added to each well and mixed, followed by addition of 10 μL of PI staining solution and an additional mixing step. The wells were incubated for 15 min at ambient temperature, protected from light, and finally subjected to confocal laser-scanning microscopy. In a parallel set of experiments, after trypsin digestion and centrifugation, the cells were stained with Annexin V-FITC/PI and immediately analyzed via flow cytometry with a FACSCalibur instrument (BD Bioscience, Franklin Lakes, NJ, USA).

Mechanism of protein delivery by IBC-Pr. HeLa cells were seeded at a density of 1.0×10^5 cells per well in 24-well plates and cultured overnight in DMEM (containing 10% (v/v) fetal bovine serum) in a CO_2 thermostat at 37°C. Subsequently, the medium was removed, and the cells were incubated with sodium azide (10 mM), sucrose (400 mM), chlorpromazine (20 μM), or cytochalasin D (10 μM) in serum-free DMEM medium at 37°C for 1 h. Another experimental group of cells was incubated at 4°C for 1 h. Next, CYC-FITC or CYC-FITC/IBC-Pr (1 μM CYC-FITC and 100 μM IBC-Pr), pre-mixed for 20 min, was added to the HeLa cells that had been exposed to the different pre-treatment conditions and incubated for 3 h at 37°C. Untreated HeLa cells were used as the control group. Confocal microscopic imaging was performed to observe the intracellular uptake of CYC-FITC. For the flow cytometry assay, the incubation procedure was the same as that described above, and the remaining steps of digestion and centrifugation were the same as those described previously. Flow cytometry was used to quantify the mean fluorescence intensity of CYC-FITC in treated HeLa cells, with the mean fluorescence intensity of the control group defined as 100%.
